# Repurposing of neprilysin inhibitor** ‘**sacubitrilat’ as an anti-cancer drug by modulating epigenetic and apoptotic regulators

**DOI:** 10.1038/s41598-023-36872-0

**Published:** 2023-06-19

**Authors:** Navanath Kumbhar, Snehal Nimal, Deeksha Patil, V. Florian Kaiser, Joachim Haupt, Rajesh N. Gacche

**Affiliations:** 1 Department of Biotechnology, Savitribai Phule Pune University, Pune, Maharashtra (MS) 411007 India; 2 Department of Microbiology, Savitribai Phule Pune University, Pune, Maharashtra (MS) 411007 India; 3 PharmAI GmbH, Tatzberg 47, 01307 Dresden, Germany

**Keywords:** Cancer, Computational biology and bioinformatics, Drug discovery, Diseases

## Abstract

Modifications in the epigenetic landscape have been considered a hallmark of cancer. Histone deacetylation is one of the crucial epigenetic modulations associated with the aggressive progression of various cancer subtypes. Herein, we have repurposed the neprilysin inhibitor sacubitrilat as a potent anticancer agent using in-silico protein–ligand interaction profiler (PLIP) analysis, molecular docking, and in vitro studies. The screening of PLIP profiles between vorinostat/panobinostat and HDACs/LTA4H followed by molecular docking resulted in five (Sacubitrilat, B65, BDS, BIR, and NPV) FDA-approved, experimental and investigational drugs. Sacubitrilat has demonstrated promising anticancer activity against colorectal cancer (SW-480) and triple-negative breast cancer (MDA-MB-231) cells, with IC_50_ values of 14.07 μg/mL and 23.02 μg/mL, respectively. FACS analysis revealed that sacubitrilat arrests the cell cycle at the G0/G1 phase and induces apoptotic-mediated cell death in SW-480 cells. In addition, sacubitrilat inhibited HDAC isoforms at the transcriptomic level by 0.7–0.9 fold and at the proteomic level by 0.5–0.6 fold as compared to the control. Sacubitrilat increased the protein expression of tumor-suppressor (p53) and pro-apoptotic makers (Bax and Bid) by 0.2–2.5 fold while decreasing the expression of anti-apoptotic Bcl2 and Nrf2 proteins by 0.2–0.5 fold with respect to control. The observed cleaved PARP product indicates that sacubitrilat induces apoptotic-mediated cell death. This study may pave the way to identify the anticancer potential of sacubitrilat and can be explored in human clinical trials.

## Introduction

Worldwide, over 19.3 million new cancer cases and 10.0 million cancer deaths have been reported in the year 2020^1^. Although therapeutic progress against cancer has been accelerated in the past decade, emerging drug resistance and off-target toxicities limit the efficacy of conventional drugs^2^. A series of intrinsic (genetic mutations, epigenetic modifications, hormones, and immune conditions) as well as non-intrinsic (chemical mutagens, toxic trace metals, and microbial infections) factors drive the progression of cancer^3^. Genetic mutations and epigenetic abnormalities alter the transcription of oncogenes and the inactivation of tumor-suppressor genes, which are responsible for cancer progression^4^. Post-translationally, the histone acetyltransferase acetylates the lysine residue on histone proteins, which results in chromatin remodeling and subsequent gene transcription in cells^3,5^. Deacetylation of lysine residues by histone deacetylases (HDACs) produces a more condensed chromatin state that alters the transcription of oncogenes and tumor-suppressor genes, which leads to progression and metastasis in cancer^6^. HDACs are Zn^2+^ and NAD^+^-dependent enzymes classified into four classes: class I (HDAC1, 2, 3, and 8), class II (HDAC4, 5, 6, 7, 9, and 10), class III (Sirtuins 1–7), and class IV (HDAC11)^6,7^.

The role of HDACs in the initiation, progression, invasion, and metastasis of various cancer subtypes has been well studied^8–10^. In addition, the contribution of HDACs has also been well documented in various pathophysiological conditions like cardiovascular diseases, diabetes, neurodegenerative disorders, inflammatory diseases, learning-memory dysfunctions, and Huntington’s disease^11,12^. Therefore, HDAC inhibitors (HDACi) such as vorinostat, panobinostat, belinostat, romidepsin, valproic acid, and tucidinostat are being widely used for the treatment of various subtypes of cancer and other diseases or disorders^13^. The HDACi reduces cell growth, proliferation, and metastasis, arrests the cell cycle at G0/G1 phases, and induces apoptosis by up-regulating pro-apoptotic (p21, p53, Bax, Bak, and Bim) and downregulating the anti-apoptotic (Bcl-2, Bcl-xL, and Mcl-1) proteins in cancer^14^. However, the cancer cells have shown varying degrees of resistance to HDACi due to intrinsic and acquired mechanisms^15^. Additionally, the HDACi are showing severe and adverse side effects, including fatigue, nausea, vomiting, anorexia, weight loss, diarrhea, dehydration, anaemia, neutropenia, asthenia, and thrombocytopenia^16^. Thus, this makes cancer treatment more difficult by reducing the overall survival rate of patients. Hence, there is an urgent need to develop more potent and selective HDACi to combat drug resistance, minimize the severe side effects, and provide a more effective treatment for cancer patients. Additionally, due to the lengthy and expensive de novo drug discovery process and drug failures in clinical trials, the drug repurposing approach is an effective way to deal with these problems^17^.

Drug repurposing (DR) is the process of finding new clinical indications for existing drugs^17–19^. Repurposing is enabled by the approved preclinical and safety studies of drugs that facilitate a faster, cheaper, and more efficient translation of drugs from bench to bedside^19^. As a result, it is gaining popularity over the traditional "de novo" drug discovery process, which is more time-consuming (13–15 years) and expensive ($2–3 billion)^18,20^. Therefore, the repurposed drugs that enter the regulatory approval pipeline are increasing every year and could account for about 30% of total approved drugs^18–20^. In the era of cancer drug discovery, the FDA-approved non-cancer drugs (Celecoxib, Itraconazole, Metformin, Raloxifene, Sildenafil, Thalidomide, and many others) have been repurposed and widely used against various cancer subtypes^21–25^.

Similarly, cardiovascular drugs including aspirin, beta-blockers (bisoprolol and propranolol), cardiac glycosides (digoxin and digitoxin), carvedilol, losartan, and statins were repurposed against cancers^25–28^. Aspirin was initially prescribed for the treatment of atherosclerotic disease, now it is repurposed against breast, colon, oesophageal, and gastric cancer by inhibiting the cyclooxygenase-1/2 enzyme and reducing prostaglandin E2 formation^29–31^. Bisoprolol and propranolol were repurposed against liver and breast cancer, respectively^32,33^. The cardiac glycosides (digoxin and digitoxin) reduced the rate of recurrence and malignant properties in the pulmonary tumor, breast and prostate cancer^34–37^. In an in-vivo study, carvedilol inhibits the proliferation and migration abilities of pancreatic cancer cells and increases the overall survival rate in mice^38^. The statins have suppressed cell proliferation in acute myeloid leukaemia, hepatocellular carcinoma, oesophageal, gastric, and prostate cancers^39–43^. Similarly, losartan has reduced cell proliferation and tumor growth by inducing apoptosis in ovarian tumors^44^. Besides these cardiovascular drugs, atenolol, sildenafil, and verapamil are currently in clinical trials (phase 2/3) against various cancer subtypes^25^. The preceding reports strongly suggest that cardiovascular drugs can be efficiently repurposed against different cancer types^27^. In addition, many antiviral, antimalarial, and antibacterial drugs have also been repurposed against cancer subtypes using in-silico, in-vitro, and in-vivo animal model studies^45–52^.

The in-silico approaches like PLIP analysis, 3D-QSAR pharmacophore modeling, machine learning, molecular docking, and MD simulations have been widely used for the repurposing of non-cancer drugs against cancer^48–50,53–59^. Currently, the PLIP analysis followed by experimental validation has achieved remarkable success in drug repurposing against cancer, neurodegenerative diseases, tuberculosis, and Chagas disease^48,56,60,61^. Similarly, the anti-malarial drug "Amodiaquine" has been repurposed against multiple myeloma by inhibiting the activity of chaperone protein (Hsp27) and reducing the chemoresistance of drugs^56^. The receptor tyrosine kinase inhibitors (Sunitinib and Crizotinib) were repurposed against Parkinson’s disease by targeting the leucine-rich repeat kinase-2^61^. A comparison of the protein–ligand binding site and PLIP analysis was used to repurpose the Fabl inhibitor against tuberculosis by inhibiting the enoyl-reductase from *Mycobacterium tuberculosis*^60^. The structure-based drug discovery and PLIP interaction fingerprint analysis were used to repurpose Bruton’s tyrosine kinase inhibitor (Ibrutinib) against cancer by inhibiting VEGFR2 and angiogenesis^48^. Similarly, molecular docking and PLIP analysis were used to repurpose the anti-diabetic compounds (Glipizide, glyburide, and gliquidone) against Chagas disease by targeting dihydrofolate reductase-thymidylate synthase^62^. Besides cancer pathophysiology, PLIP was also used to repurpose the drugs against COVID-19 by targeting the RNA-dependent RNA polymerase and SARS-CoV2-main protease (Mpro)^63–67^.

The literature survey may help to design an effective strategy to treat various human diseases by identifying new clinical indications for the existing FDA-approved drugs using PLIP analysis followed by their experimental validations. Moreover, considering the critical role of HDACs in the progression, invasion, and metastasis of cancer, herein we have attempted to repurpose small non-cancer drugs as anticancer agents by targeting HDACs. The PLIP screening was used to identify possible HDACi (Sacubitrilat) and its validation was done using in-vitro studies. The outcome of the present study demonstrated the promising anti-cancer activity of sacubitrilat against colorectal (SW-480) and TNBC (MDA-MB-231) cells by ROS production and inducing apoptotic-mediated cell death in SW-480 colorectal cancer cells. The sacubitrilat modulated the expression of HDAC isoforms at transcriptomic as well as proteomic levels. It also reduced the expression of anti-apoptotic (Bcl2 and Nrf2) proteins whereas up-regulated the expression profile of pro-apoptotic (p53, Bax, Bid, and cleaved Parp) proteins in SW-480 cells. Our in-silico and in-vitro studies supported the repurposing of neprilysin inhibitor ‘Sacubitrilat’ as a potential anti-cancer agent against colorectal cancer. The results of in-silico and in-vitro analysis may pave the way to elucidate the anti-cancer potential of sacubitrilat against colorectal and TNBC cancer cell lines. However, further pre-clinical and clinical studies are required to ascertain the mechanistic role of sacubitrilat against colorectal cancer.

## Materials and methods

The protein–ligand interaction profile analysis of vorinostat (SAHA, SHH) and panobinostat (LBH) with HDACs and LTA4H.

The protein–ligand interaction profiles of FDA-approved HDAC inhibitors such as vorinostat (SAHA, SHH) and panobinostat (LBH) in complexes with HDACs and Leukotriene A4 hydrolase (LTA4H) were analyzed using the PLIP tool on the PDB structures (1C3S, 1T69, 1ZZ1, 3C0Z, 4BZ6, 4LXZ, 4QA0, 4QA2, 4R7L 5EEI, and 5EF8^68^. The interaction profiles for each of these structures were generated with PLIP, representing the non-covalent interactions between the inhibitors and the targets. The most common interactions identified by PLIP between SAHA/panobinostat and HDACs/LTA4H are hydrogen bonds, hydrophobic interactions, π-stacking, water bridges, and metal-coordination interactions. Bit vectors were used to encode the interaction fingerprints, such that each bit represents a feature defined by the combination of two non-covalent interactions within an angle and distance range. This value is set to 1 if the feature is present in the binding or to 0 if it is not^48,56^. The resulting PLIP fingerprints were screened against the PLIP fusion fingerprint database, which covers the full PDB, to identify other protein–ligand complexes with a similar binding pattern. Further, the obtained hit compounds were sorted by removing biologically irrelevant entries using the BioLiP database^69^ and were subjected to molecular docking studies against LTA4H and HDACs.

### Molecular docking studies of PLIP-screened hit compounds against LTA4H and HDAC6

Molecular docking was performed to select the promising hit compounds with potent HDAC inhibitory activity by using Autodock 4.2 software^70^. The SAHA and panobinostat were used as reference drugs while performing the docking study. The starting geometry of hit compounds, SAHA and panobinostat, were retrieved from the Protein Data Bank and geometrically optimized by the semi-empirical RM1 method using Spartan’14 software (Wavefunction, Inc., Irvin, CA). The receptor structures of LTA4H (4R7L) and HDAC6 (5EF8) were retrieved from the Protein Data Bank and used for the molecular docking study. The receptor structures were refined by removing non-polar hydrogen atoms, adding Kollman united atom charges, and adding polar hydrogen atoms using the Autodock wizard. Gasteiger charges and hydrogen atoms were added to the hit compounds, SAHA and panobinostat. The AutoGrid module was used to calculate the grid map and centered on the catalytic sites of receptor structures in such a way that it would cover the hit compounds, SAHA and panobinostat. For LTA4H/HDACs, the grid size was set to 48 Å × 48 Å × 48 Å with a grid spacing of 0.375. The step size for translation was set to 1, and the maximum number of energy evaluations was set to 2,500,000. The 100 runs of docking were performed for receptor-ligand molecules with a maximum number of 2,70,000 LGA operations that were generated on a single population of 150 individuals. The operator weights for a crossover (0.80), mutation (0.02), and elitism (1) were maintained as default parameters. Docked complexes with the lowest energy stable conformation were analyzed for molecular interactions, and the PLIP web server was used to create the pictorial presentation.

### In-vitro cell culture studies

Major chemicals: The 3-(4,5-Dimethylthiazol-2-yl)-2,5-diphenyltetrazolium bromide (MTT) was purchased from Himedia. Dulbecco's Modified Eagle Medium (DMEM) high glucose media was purchased from Gibco, and 5-(and-6)-chloromethyl-2′,7′-dichlorofluorescein diacetate acetyl ester (CM-H_2_ DCFDA) was procured from Sigma. Annexin V-FITC apoptosis kit was used from Biolegend. The apoptosis-specific antibodies such as PARP and NRF2 (cell signaling technology), Bax, Bad, Bid, and Bcl2 were purchased from Santacruz Biotechnology. HDAC isoform antibodies were purchased from Cell Signaling Technology, and HRP-conjugated secondary mouse and rabbit antibodies were purchased from Santacruz Biotechnology.

### Screened Hit compounds

Out of the five screened hit compounds, only sacubitrilat (6LD, LBQ657) is commercially available at Sigma Aldrich. Therefore, sacubitrilat and SAHA were purchased from Sigma Aldrich, and the stock solution was prepared in sterile-filtered dimethylsulfoxide (DMSO) and stored at − 20 °C.

### Cell lines and culture

The human colon cancer (SW480) and triple-negative breast cancer (MDA-MB-231) cell lines were purchased from the National Centre for Cell Science (NCCS), a national facility for providing animal cell lines in Pune (MS), India. The cell lines were cultured in DMEM medium supplemented with 10% FBS, penicillin–streptomycin (50 unit/mL; Invitrogen), and were maintained at 5% CO_2_ and 37 °C.

### Determination of cell viability by MTT assay

The effect of sacubitrilat and SAHA (reference drug) on the cell viability of colon and TNBC cancer cells was assessed using an MTT cell proliferation assay. The MTT assay protocol was adopted from earlier studies^71–73^. In brief, the SW-480 and MDA-MB-231 cells were seeded at a cell density of 1 × 10^3^ cells/per well into a 96-well culture plate. The effects of sacubitrilat and SAHA on the viability of colon and TNBC cells were tested at different concentrations (10–70 µM), and the cells were incubated for 48 h. After the treatment, the cell culture medium was replaced with 100 μl of MTT reagent [3-(4,5-dimethylthiazol-2-yl)-2,5-diphenyltetrazolium bromide] and incubated at 37 °C for 4 h. The mitochondrial reductase from metabolically active cells reduced the water-soluble yellow tetrazolium dye into an insoluble formazan crystal. Then, these crystals were solubilized in 100 µL of DMSO, which on the addition of solvent turns into a purple color. The formation of the formazan product was measured spectrophotometrically at 570 nm using Hidexsense multimode plate reader. The data is plotted as the concentration of drugs versus the percentage of cell proliferation.

### Cell migration assay using scratch wound healing

The scratch assay was performed as described in our earlier reports^72,73^. Briefly, the colon cancer cells were seeded in 24 well plates (1 × 10^4^ cells/well) and grown till confluency. Then scratches were made by using sterile pipette tips in all wells and treated with sacubitrilat and SAHA for 48 h. The microscopic images of scratch wound healing of control, sacubitrilat and SAHA treated were captured at 0 h, 24 h, and 48 h using a phase contrast microscope. The images were processed for distance measurement of wound healing using Zen software. The graphs were plotted for time versus distance of scratch wound healing for control and treated cells.

### Role of sacubitrilat in the generation of reactive oxygen species using DCFDA Assay

The role of sacubitrilat and SAHA in the generation of intracellular reactive oxygen species** (**ROS) has been investigated by performing a DCFDA assay using the protocol described in our previous study^72^. In brief, the SW-480 cells were seeded in 96 well plates (1 × 10^3^). The cells were treated with sacubitrilat and SAHA for 48 h. After the treatment, the cells were washed with PBS (pH 7.4) and exposed to DCFHDA (10 μM) stained for 20 min followed by washing with PBS. The generation of ROS was measured using a Hidexsense multimode spectrophotometer and images for the uptake of 2′,-7′-dichlorofluorescein (DCF) were captured using live cell imaging (Zeiss).

### Cell cycle analysis using fluorescence-activated cell sorting (FACS) and Live cell imaging analysis

To investigate the effect of sacubitrilat on the regulation and phase distribution of the cell cycle in SW-480 cells, we performed propidium iodide (PI) staining using flow cytometry analysis^72,73^. The SW-480 (1 × 10^6^) cells were seeded in 35 mm plates and treated with sacubitrilat and SAHA for 48 h. After treatment, cells were stained with PI (50 μg/mL). The regulation of cell cycle phase distribution was determined using FACS. The BD Cell Quest Pro software was used to analyze the results. Live cell imaging of PI uptake was done using Zeiss live cell imager.

### Role of sacubitrilat in the induction of apoptosis in SW480 cells

To investigate the role of sacubitrilat in the induction of apoptosis in SW-480 cells, we have performed the Annexin-V-FITC assay using FACS analysis^71–73^. The assay was carried out using Annexin V-FITC Apoptosis Detection Kit (Biolegend). The experimental protocol was employed as per the manufacturer’s instructions. In brief, the cells were seeded at a density of 1 × 10^6^ in 35 mm plates and treated with sacubitrilat (14.07 μg/mL) and SAHA (0.570 μg/mL). After treatment, the cells were washed with PBS (1X) and fixed in 70% ice-cold methanol at − 20 °C for 2 h. Then, the Annexin V-FITC (50 μg/mL) was added followed by PI (50 μg/mL) in control and treated cells. The DNA content of stained nuclei was captured using a flow cytometer (BD Bioscience) and analysis of results was carried out using BD Cell Quest Pro software.

### Quantitative expression analysis of epigenetic regulators (HDACs) using qRT-PCR

The effect of sacubitrilat and SAHA on the expression of epigenetic regulators (HDACs) was elucidated in SW-480 cells by performing the qRT-PCR. The SW-480 cells were grown to 70% confluency and treated with sacubitrilat and SAHA at IC_50_ concentration. The protocol for measuring the expression of mRNA was carried out as per the earlier reported method^74^. In brief, total RNA from control and treated samples were isolated using TRIZOL (Invitrogen USA) reagent. The obtained RNA samples were reconstituted in nuclease-free water and were quantified using SpectroStar-NanoBMG, Labtech. The cDNA reverse transcription kit (iScript, Biorad) was used for the synthesis of cDNA from total isolated RNA as per the manufacturer's protocol. The temperature profile was 25 °C for 5 min, 46 °C for 20 min, and 95 °C for 5 min were used for the reverse transcription using Mycycler Thermal Cycler, Biorad. The synthesized cDNA was used for real-time PCR using SYBR green (CFX96 Real-time System, Biorad) along with HDACs and GAPDH-specific primers. The GAPDH housekeeping gene was used for the data normalization. Fold changes in the mRNA expression levels of epigenetic regulators were analyzed using the 2^−ΔΔCT^ method^75^.

### Quantitative protein expression profiling of epigenetic and apoptosis regulators using western blot analysis

Western blot analysis (WBA) was carried out to validate the mRNA expression profile of epigenetic regulators. Similarly, pro-apoptotic markers (BAX, BID and PARP) and anti-apoptotic BCL2 markers were studied in SW-480 after the treatment of sacubitrilat and SAHA. The WBA was carried out as per the previously described method^72,73^. In brief, the SW-480 cells (2 × 10^5^) were seeded in 60 mm plates and treated with sacubitrilat and SAHA. After treatment, the cells were washed with PBS, scraped, pelleted, and lysed in RIPA buffer (Thermofisher) containing a protease inhibitor cocktail (Roche). After incubation for 30 min on ice, the cell lysates were centrifuged at 12,000 rpm for 45 min at 4 °C. The supernatants were collected and protein concentrations were measured using the Bradford reagent. The total cell proteins (30 μg/mL) were electrophoresed on 7.5–12% SDS-PAGE gel using a Biorad-All blue protein ladder. After the complete resolution of proteins, the gels were transferred onto a polyvinylidene fluoride (PVDF) membrane. The membranes were blocked using skimmed milk prepared in Tris-buffered saline containing 0.1% Tween-20 (TBST) for 1 h. Followed by three consecutive TBST washes the membranes were incubated with an optimal dilution of the desired primary monoclonal antibodies at 4 ºC overnight. The membrane was washed three times with TBST and incubated with an optimal dilution of the appropriate secondary antibodies conjugated with horseradish peroxidase (HRP) for 2 h at room temperature. The membranes were exposed to ECL advansta detection reagents and the specific protein band was digitalized using the system Amersham Gel-imager-680.

### Statistical analysis

All the experiments were done in triplicates. The statistical analysis was done by GraphPad-PRISM version 5.01 using one-way ANOVA. The error bar represents mean ± SD derived from three independent replicates. The *, ** and *** denotes *p*-values ≤ 0.05, ≤ 0.01 and ≤ 0.001, respectively.

## Results

### Protein–ligand interaction profile analysis of vorinostat (SHH) and panobinostat (LBH)

The crystal structures containing vorinostat and panobinostat as co-crystals were analyzed for protein–ligand interactions profile using the PLIP server. The 10 PLIP profiles of SAHA in complexes with HDAC2, HDAC4, HDAC6, HDAC7, HDAC8, and LTA4H were analyzed and given in Table 1.

**Table 1:** Protein–ligand interaction profiles of vorinostat (SAHA, SHH) and panobinostat with HDAC2, HDAC6, HDAC7, HDAC8, HDACs homolog, and LTA4H.

PDB ID	Hydrogen bond	Hydrophobic Interactions	Metal interactions	Salt bridges	Water bridges
Vorinostat (SAHA, SHH)					
4LXZ (HDAC2)* Homo sapiens*	Asp104 (2.0/165.67) Tyr308 (1.58/160.71)	Phe155 Phe210	Asp181(2.00) His183 (2.07) Asp269 (1.90)		SHH-W-Gln265 SHH-W-Tyr308
4QA0 (HDAC8)* Homo sapiens*	Tyr306 (1.65/163.09)	Phe152,Phe208, Pro273, Tyr306	Asp178 (1.90) His180 (2.16) Asp267 (2.08)	Asp178 Asp267	SHH-W-Asp101
4QA2 (HDAC8)* Homo sapiens*	Tyr306 (1.62/170.79)	Phe152,Phe208, Pro273, Tyr306	Asp178 (2.00) His180 (2.07) Asp267 (2.07)		
4BZ6 (HDAC8)* Schistosoma mansoni*	His292 (2.77/148.04)	Phe216,Pro291, Tyr341	Asp186 (2.06) His188 (2.12) Asp285 (1.97)		SHH-W-Lys20 SHH-W-Tyr341
3C0Z (HDAC7)* Homo sapiens*	His670 (1.76/134.23)	Phe679, Phe738	Asp707 (2.05) His709 (2.03) Asp801 (2.04)		SHH-W-Gly842
1C3S (HDAC homolog)* Aquifex aeolicus*		Pro22, Phe111	Asp168 (1.93) His170 (2.16) Asp258 (1.91)	Asp168 Asp258	
1ZZ1 (HDAC like)* Alcaligenaceae bacterium FB188*	Asp98 (3.14/109.01) Gly151 (3.37/123.23)	Leu21, Ile100, Phe152, Phe208, Phe341	Asp180 (2.14) His182 (2.16) Asp268 (1.97)	Asp180 Asp268	
4R7L (Leukotriene A4 Hydrolase)* Homo sapiens*	Gln136 (2.76/105.31) π-stacking_Phe314	Gln136,Tyr267, Trp311,Phe314, Leu369, Pro374 Tyr378	His299 (2.07) Glu318 (2.93)		SHH-W-Pro374 SHH-W-Asp375
5EEI (HDAC6) *Danio rerio*	His573 (2.49/167.65) His574 (1.91/140.31) Tyr745 (1.74/174.81)	Pro464,Phe583, Phe643,Leu712	Asp612 His614 Asp705		
1T69 (HDAC8)* Homo sapiens*	His180 (3.41/127.87)	Tyr100,Phe152, Phe208	Asp178 (2.78) His180 (1.79) Asp267 (1.91)	Asp178 Asp267	
Panobinostat (LBH)					
5EF8 (HDAC6)* Danio rerio*	π-stacking_Phe643 π-cation_His463	Phe583, Leu712	Asp612 (2.09) His614 (2.19) Asp705 (1.95)		

**Figure 1:**
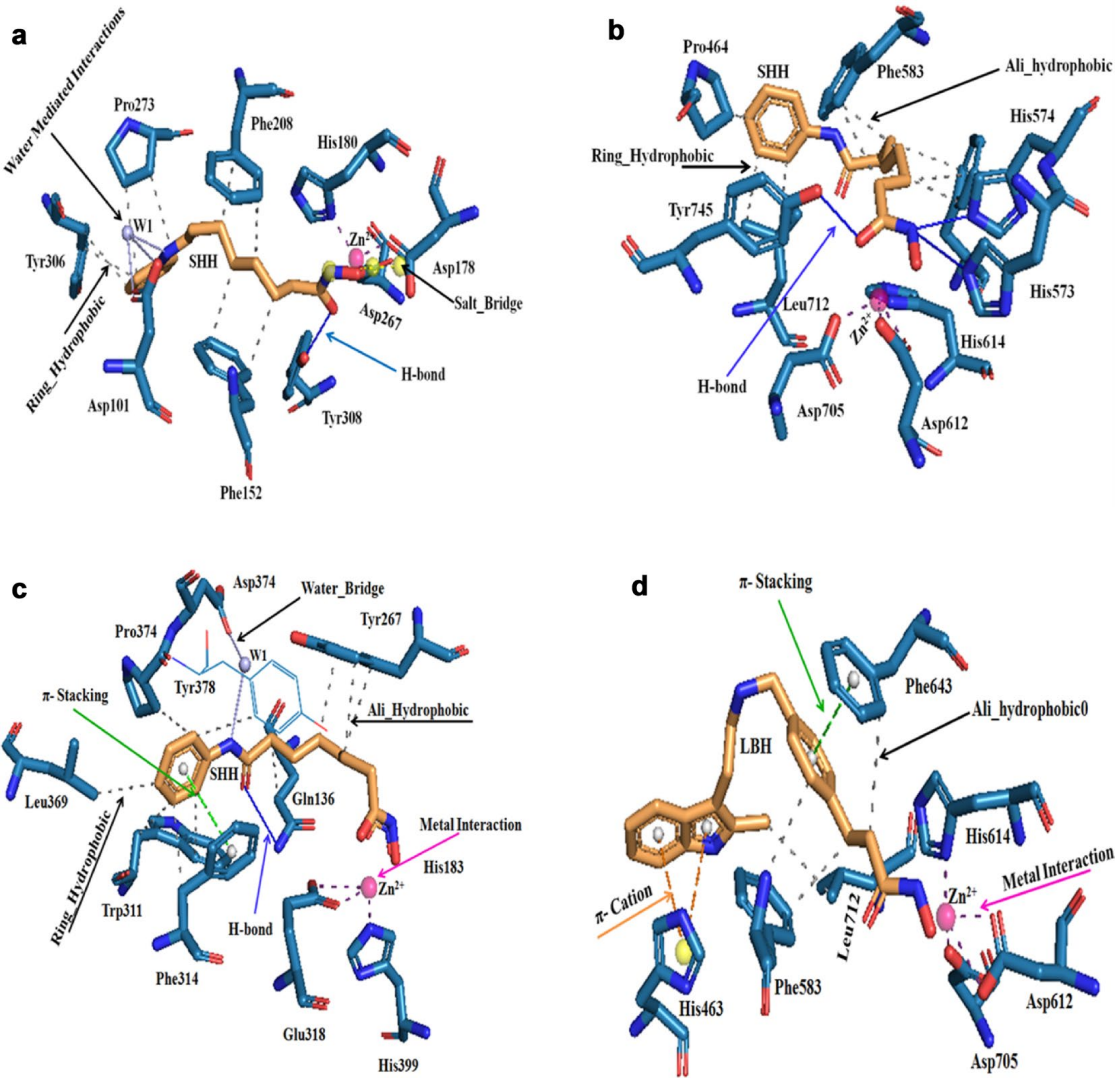
Protein–ligand interaction profile of SAHA (SHH) and panobinostat with HDACs; (**a**) PLIP profile of SAHA with 5EEI (HDAC6), (**b**) PLIP profile of SAHA with 4QA0 (HDAC8), (**c**) PLIP profile of SAHA with 4R7L (LTA4H), and (**d**) PLIP profile of panobinostat (LBH) with 5EF8 (HDAC6).

The PLIP profiles between SAHA and HDACs contain hydrogen bonding, hydrophobic, water bridges, and metal coordinate interactions. Whereas, the PLIP profile between SAHA and LTA4H contains one additional π-stacking interaction, which was not observed in the PLIP profiles of SAHA with HDACs (Table 1). Therefore, the common PLIP profiles of SAHA with HDAC6 (5EEI), HDAC8 (4QA0) and LTA4H (4R7L), and the PLIP profile of panobinostat with HDAC6 (5EF8) were selected for the screening of PLIP fusion fingerprint database (Fig. 1).

This database contains the interaction patterns (IPs) for 170,351 crystal structures derived from the Protein Data Bank. After screening, a total of 1393 biological complexes with 114 co-crystal inhibitors (*p*-value cut-off of > 1.00E−03) were obtained which contain similar PLIP profiles as observed in SAHA/LTA4H and panobinostat/HDAC6. However, the screening of the PLIP profile between SAHA and HDAC6/HDAC8 did not result in significant hits. The biologically irrelevant entries from the obtained hit compounds were removed using the BioLiP database, which resulted in 75 hits. Out of 75 hits, 34 hits were matched with the PLIP of SAHA and LTA4H and 41 hits with panobinostat and HDAC6. The 75 hits were classified into FDA-approved, experimental, investigational drugs using the Therapeutic Target Database^76^. A total of 17 drugs; eight hits (Sacubitrilat, 6TJ, 7CA, B65, BDS, BIR, CRI, and HPI) resulted against the SAHA/LTA4H profile, and nine hits (792, AP5, AXA, DPS, NPV, T5A, TXC, YE7, and Z5A) resulted against the PLIP profile of panobinostat/HDAC8. These hit compounds were subjected to molecular docking studies against LTA4H and HDAC6 targets.

### Molecular docking studies of hit compounds against LTA4H and HDAC6

**Figure 2:**
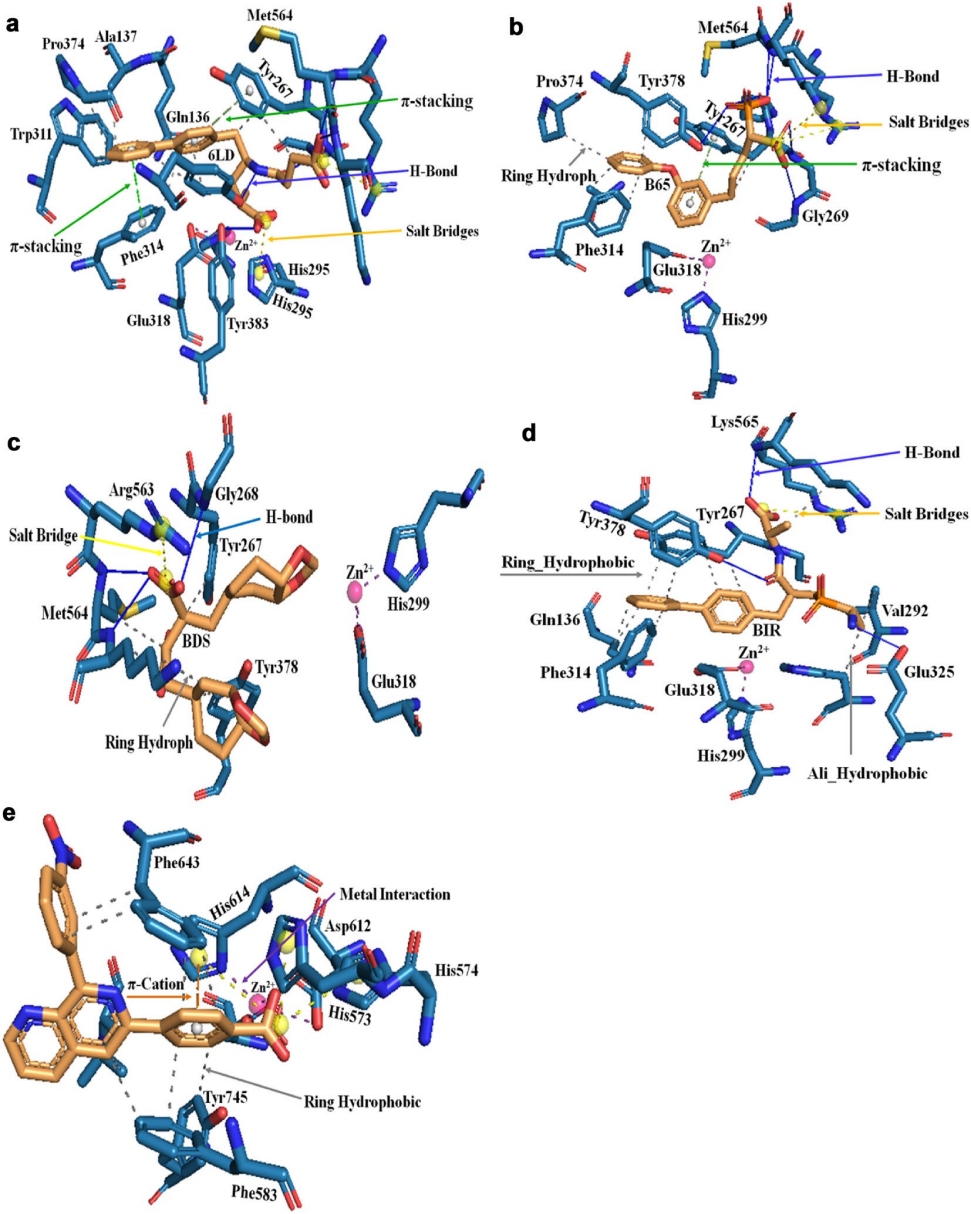
The protein–ligand interaction profiles of docked complexes of screened hit compounds with LTA4H and HDAC6; (**a**) Sacubitrilat (6LD)-4R7L, (**b**) B65-4R7L, (**c**) BDS-4R7L, (**d**) BIR-4R7L and (**e**) NPV-5EF8.

The molecular docking studies of screened hit compounds against the LTA4H and HDAC6 were performed by using SAHA and panobinostat as reference drugs. The screening of the PLIP fusion fingerprint database gave significant hits for SAHA/LTA4H and panobinostat/HDAC6 PLIP profiles. Therefore, the docking results of hit compounds against LTA4H and HDAC6 are discussed here. Molecular docking of eight hits with LTA4H resulted in four hits (Sacubitrilat, BIR, B65, and BDS) which yielded the lowest energy stable conformations as compared to reference drug SAHA (Fig. 2 and Table 2). Figure 2 depicts the molecular interactions such as hydrogen bonds, π-stacking, hydrophobic, salt bridges, and metal-coordination interactions from the favored docked complexes of hit compounds with LTA4H. Also, the binding energies and geometrical parameters including the bond distances and bond angles for hydrogen bonding and metal-coordination interactions from the favorable docked complexes are given in Table 2. The sacubitrilat produced the lowest energy stable docked complex with LTA4H (− 10.39 kcal/mol). In this docked complex, sacubitrilat is positioned in the deep catalytic pocket of LTA4H, where it coordinated with Zn^2+^ by maintaining a 3.4 Å distance (Fig. 2a). The carbonyl oxygen and amide nitrogen of the side chain of sacubitrilat were involved in strong hydrogen bonding interactions with Gly269 and Tyr378 by maintaining 1.94 Å and 2.25 Å bond distances, respectively (Table 2).

**Table 2:** Molecular interactions such as hydrogen bonds, π-stacking, hydrophobic, metal and salt bridges from the favorable docked complexes of sacubitrilat (6LD), B65, BDS and BIR with LTA4H (4R7L), and NPV with HDAC6 (5EF8).

Name of hit compound	Hydrogen bonds (Bond distances and Angles)	Salt bridge	Hydrophobic interactions	Metal interactions	Binding energy (Kcal/mol)
SAHA/4R7L (Reference)	Gln136 (2.76/105), π-stackingPhe314		Trp311,Leu369, Pro374, Tyr378	SHH-Zn^2+^ (2.1) SHH-Zn^2+^ (2.0)	− 9.56
Sacubitrilat (6LD)	Gly268 (2.89/131), Gly269 (1.94/175), Tyr378 (2.25/151), Tyr383 (1.73/170), Met564 (3.0/100), Lys565 (1.98/156), π-stacking_Phe314, π-stacking_Tyr267	His295, Arg563	Gln136, Ala137, Tyr267, Trp311, Phe314, Pro374, Tyr378	6LD-Zn^2+^ (3.4) 6LD-Zn^2+^ (3.5)	− 10.39
BIR	Gly268 (2.63/154), Glu325 (2.20/152), Tyr378 (2.72/102), Lys565 (2.66/121),	Arg563	Gln136, Tyr267, Val292, His295, Phe314, Tyr378, Lys565		− 10.38
B65	Pro266 (3.79/100), Gly268 (3.00/125), Gly269 (3.1/127), Tyr378 (2.93/121), Met564 (3.54/102), Lys565 (2.31/146), π-stacking_Tyr267	Arg563 Lys565	Ala137, Tyr267, Phe314, Pro374, Tyr378		− 9.92
BDS	Gly268 (2.51/129), Met564 (2.72/104), Lys565 (2.1/172)	Arg563	Tyr267, Tyr378, Met564, Lys565	BDS-Zn^2+^ (3.1) BDS-Zn^2+^ (3.5)	− 9.58
Panobinostat (LBH)/5EF8 (Reference)	π-stacking_Phe643 π-cation_Phe463	Asp612 Asp705	Phe583, Leu712	LBH-Zn^2+^ (2.0) LBH-Zn^2+^ (2.6)	− 6.21
NPV	π-cation_His614	His573, His574, His614	Phe583, Phe643, Leu712, Tyr745	NPV-Zn^2+^ (2.3) NPV-Zn^2+^ (3.3)	− 7.99

In addition, the terminal carboxyl group of sacubitrilat participated in hydrogen bonding interactions with Gly268, Met564, and Lys565 residues from the catalytic pocket of LTA4H (Fig. 2a). Another carboxyl end of sacubitrilat was forming hydrogen bonding interactions with Tyr383 by adopting a 1.73 Å bond distance. The two phenyl rings of sacubitrilat were involved in π-stacking interactions with the catalytic residues Tyr267 and Phe314 of LTA4H. The observed hydrogen bonding and hydrophobic interactions could provide structural stability to the sacubitrilat-LTA4H complex. The bifurcated carboxyl ends of sacubitrilat also formed salt bridges with His295 and Arg563 residues. The additional stability of the sacubitrilat-LTA4H docked complex was expected from the hydrophobic interactions between Gln136, Ala137, Tyr267, Trp311, Phe314, Pro374, and Tyr378 residues from the active site pocket of LTA4H (Table 2). By allowing suitable conformational space for phenyl rings and bifurcated side chains, all non-covalent interactions provided structural stability to the sacubitrilat in the catalytic pocket of LTA4H. Similar hydrogen bonding, π-stacking, Zn^2+^ coordination, and hydrophobic interactions were observed in the favorable docked complexes of B65, BDS and BIR with LTA4H (Fig. 2b–d, and Table 2). All these non-covalent interactions are comparable to the molecular interactions of the reference drug SAHA with LTA4H and HDAC isomers (Fig. 1, Tables 1 and 2). These docking results help to understand the mechanism of catalytic inhibition of LTA4H and HDAC isomers by sacubitrilat, B65, BDS and BIR.

Similarly, the NPV showed the highest binding affinity toward HDAC6 (5EF8) and yielded the lowest energy (− 7.99 kcal/mol) stable complex as compared to the FDA-approved drug panobinostat (− 6.21 kcal/mol) (Fig. 2e). Here also, we noticed a similar protein–ligand interaction pattern between the panobinostat and NPV with 5EF8 (Table 2). Out of these five (Sacubitrilat, B65, BDS, BIR, and NPV), only sacubitrilat is commercially available. Therefore, we have validated the anticancer potential of sacubitrilat using in-vitro studies.

### In-vitro studies of sacubitrilat against cancer cells

#### Effect of sacubitrilat on cell viability of SW-480 and MDA-MB-231 cells using MTT assay

**Figure 3:**
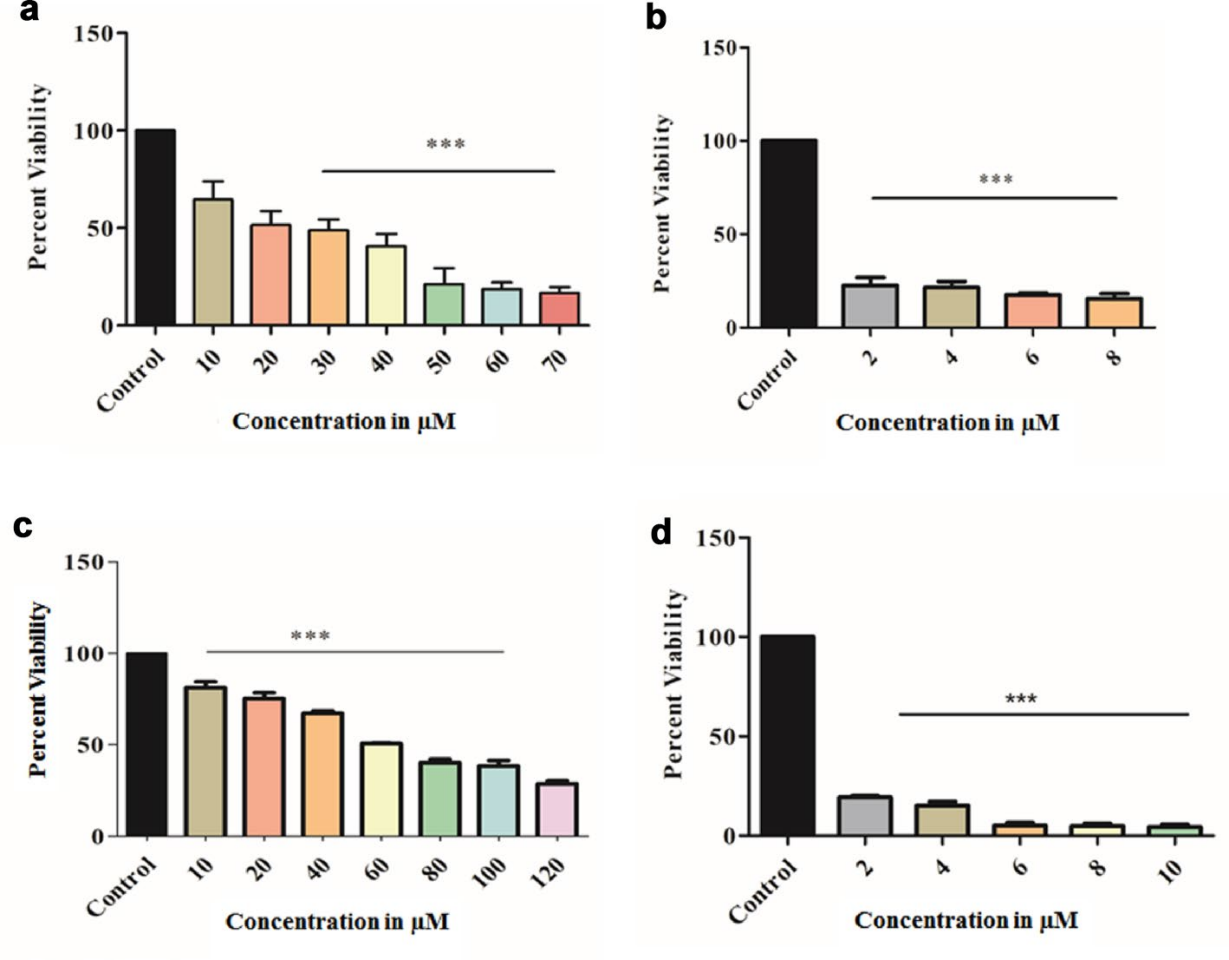
(**a**) Effect of sacubitrilat on cell viability of SW-480, (**b**) Effect of SAHA on cell viability of SW-480, (**c**) Effect of sacubitrilat on cell viability of MDA-MB-231, and (**d**) Effect of SAHA on cell viability of MDA-MB-231.

The effect of sacubitrilat and SAHA on the cell viability of colon cancer (SW-480) and triple-negative breast cancer (MDA-MB-231) cells was studied using the MTT assay (Fig. 3). Figure 3 depicts the inhibition of cell proliferation of SW-480 and MDA-MB-231 cells in a dose-dependent manner. The increasing concentrations of sacubitrilat and SAHA decreased the viability of SW-480 and MDA-MB-231 cells (Fig. 3a–d). The calculated IC_50_ values for sacubitrilat on SW-480 and MDA-MB-231 are 36.71 µM/mL (14.076 µg/mL) and 60.04 µM/mL (23.02 µg/mL).

**Figure 4:**
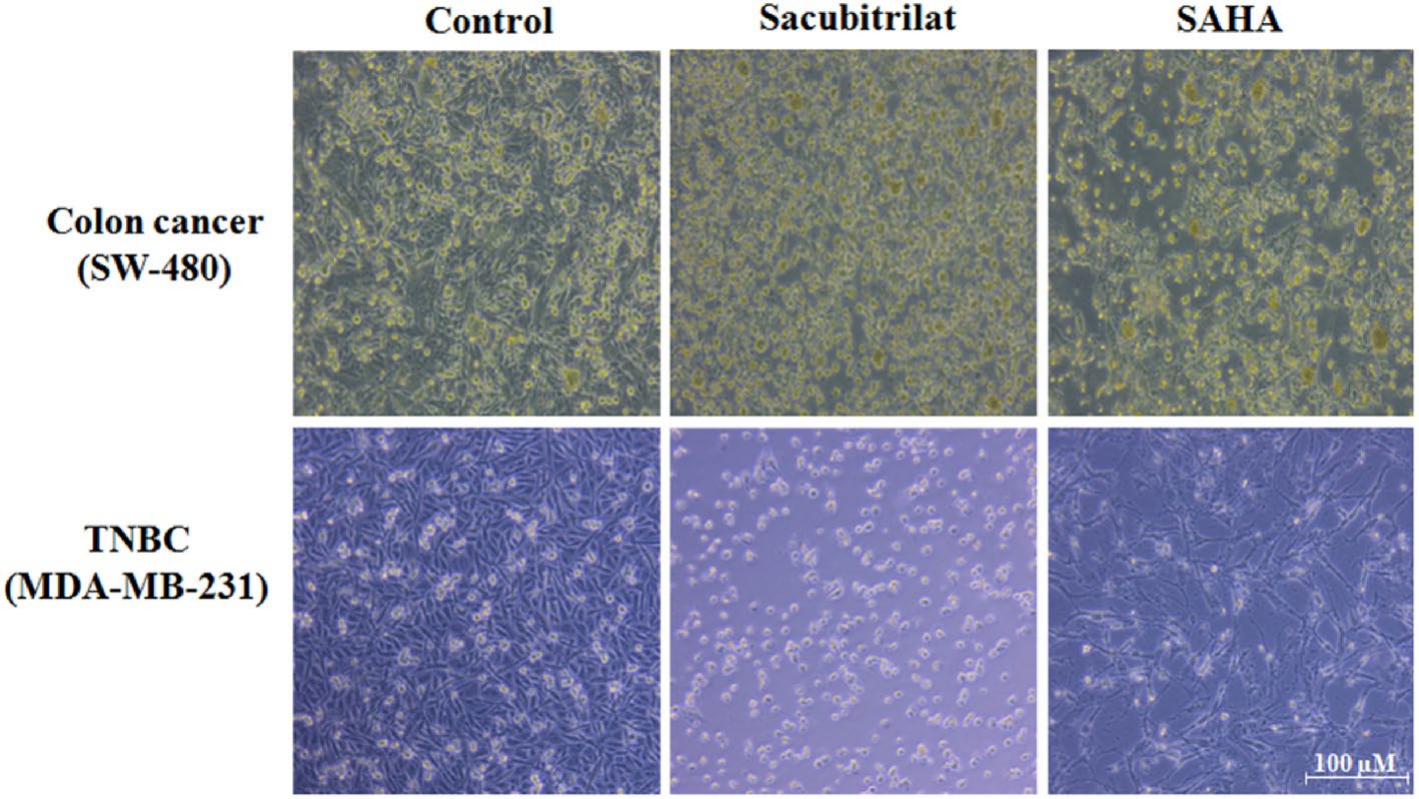
Effect of sacubitrilat and SAHA on the morphology of SW-480 and MDA-MB-231 cells.

Sacubitrilat and SAHA induce adverse morphological changes in colon cancer and TNBC cells at their IC_50_ concentration (Fig. 4). The SW-480 cells are more sensitive to the sacubitrilat drug treatment as compared to MDA-MB-231 cells. Therefore, we have explored the role of sacubitrilat on colon cancer (SW-480) cells in detail.

#### Cell migration using a scratch wound healing assay

**Figure 5:**
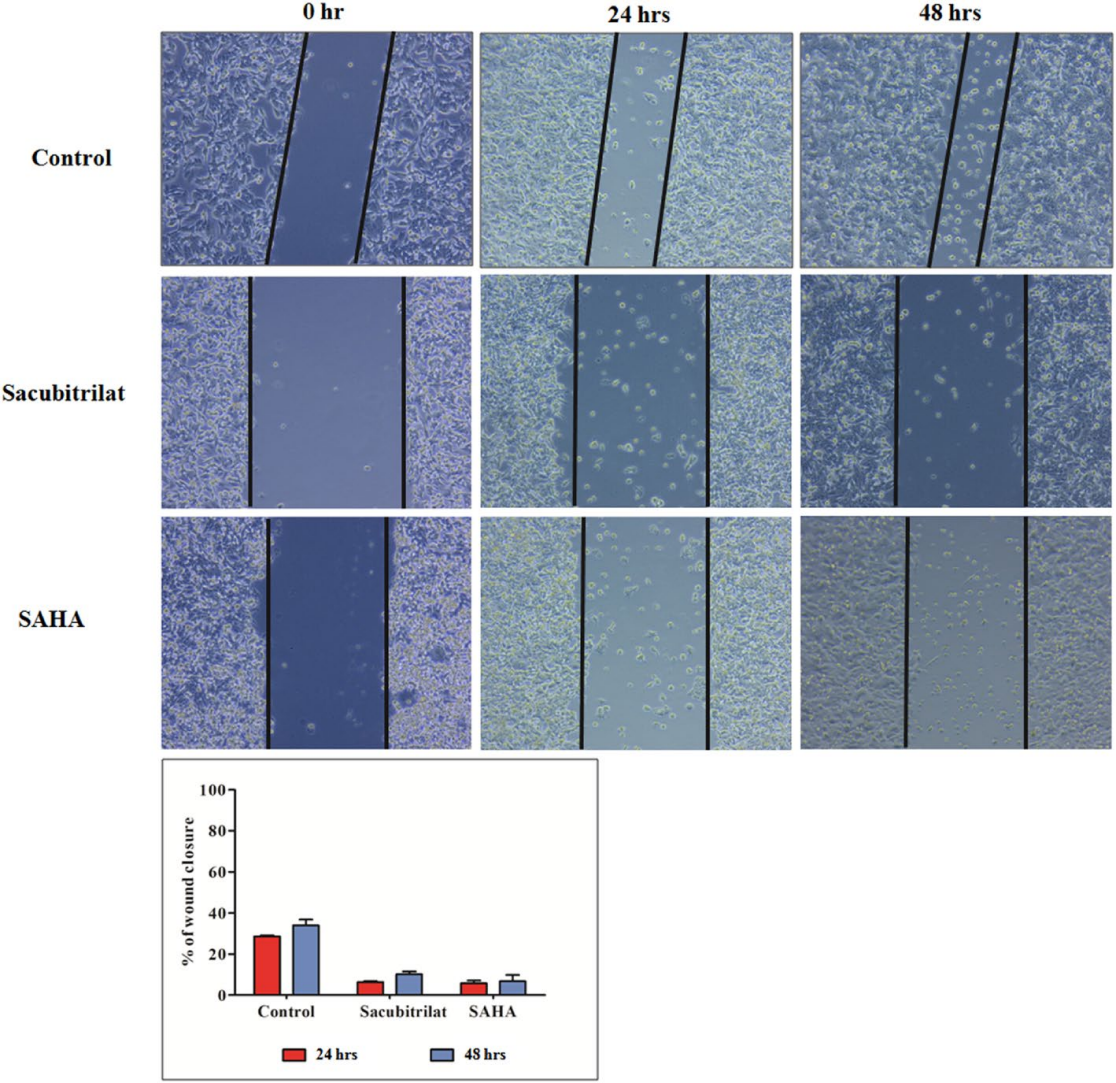
Effect of sacubitrilat and SAHA on cell migration ability of SW-480 cells using wound healing assay; (**A**) Cell migration in control (SW-480) cells, (**B**) Sacubitrilat inhibiting the migration ability of SW-480 cells, (**C**) SAHA inhibiting the migration ability of SW-480 cells.

The wound healing assay is a commonly used method to investigate cell migration and metastasis in cancer. Herein, we have demonstrated the anti-metastatic potential of sacubitrilat and SAHA by inhibiting the migration ability of SW-480 cells using a wound healing assay (Fig. 5).

Cell migration was observed in control SW-480 cells. On the other hand, sacubitrilat inhibited the migration ability of SW-480 cells. The wound healing results are comparable to the reference drug SAHA. These results indicated that sacubitrilat may act as a good anticancer agent by decreasing the metastatic potential of cancer cells.

#### Sacubitrilat generates reactive oxygen species in SW-480 cells

**Figure 6:**
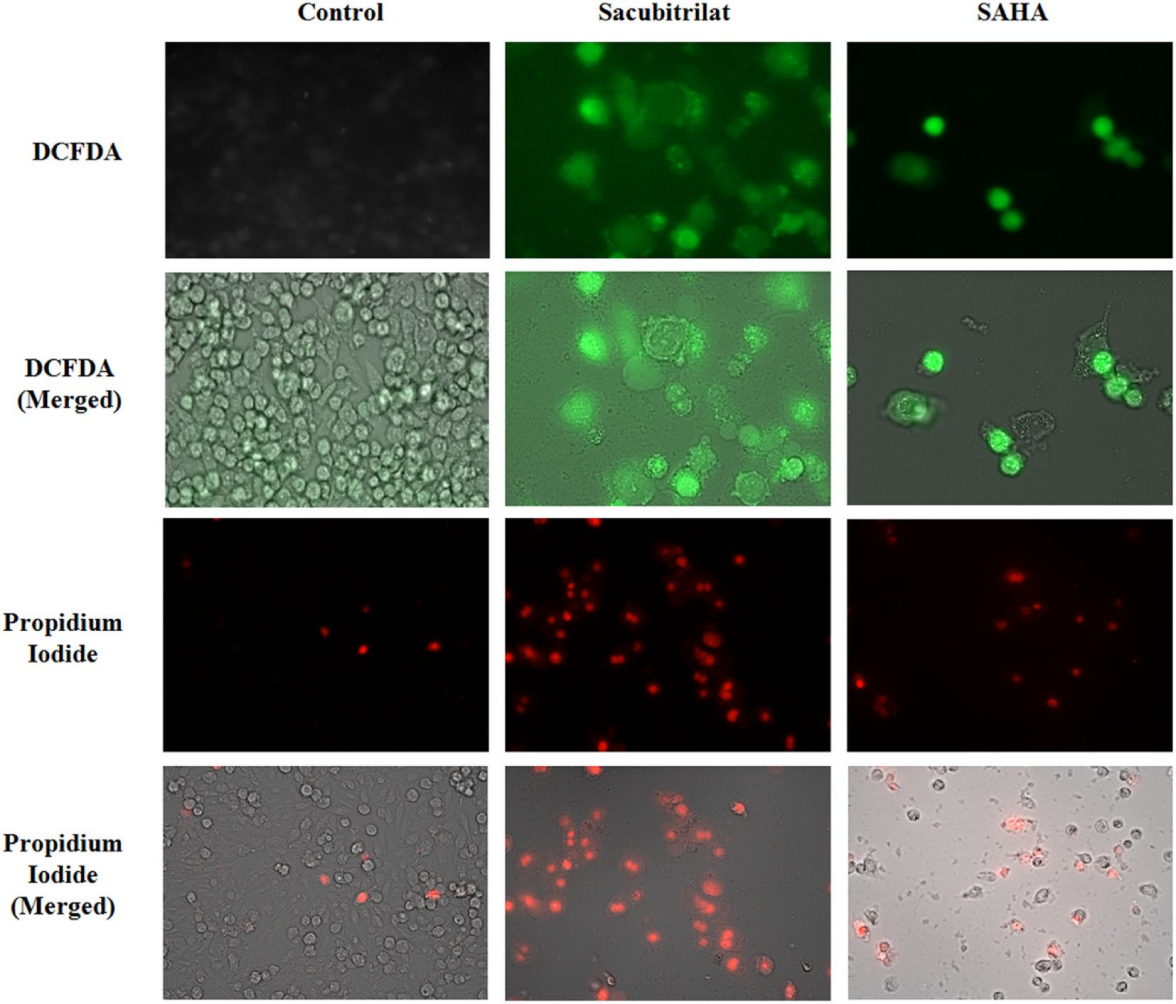
Generated reactive oxygen species in SW480 cells after 48 h of treatment of sacubitrilat and SAHA. Propidium iodide staining for control and treated SW-480 cells (Sacubitrilat and SAHA).

**Figure 7:**
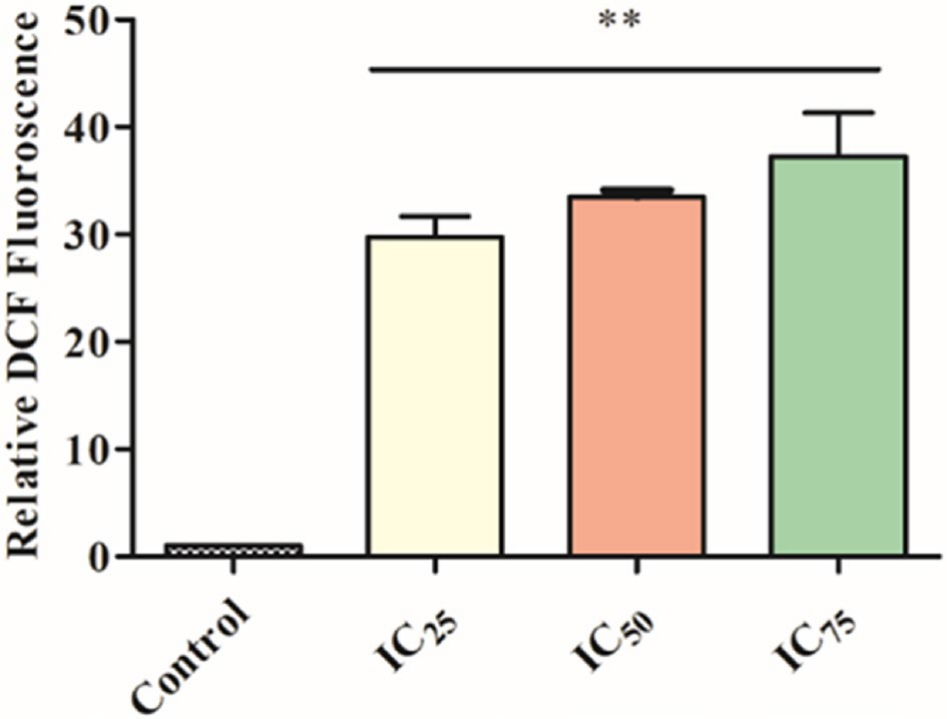
The reactive oxygen species generation in SW-480 cells after the treatment of sacubitrilat at increasing drug concentrations.

The generation of intracellular reactive oxygen species (ROS) in SW-480 cells after the treatment of sacubitrilat was analyzed using a colorimetric assay and live cell imaging system (Fig. 6). DCFDA staining is one of the most important techniques widely used to measure the redox state of cells. The DCFDA uptake in sacubitrilat-treated cells was higher which signifies that SW-480 cells produced a high level of ROS as compared to control cells (Fig. 6). This alters redox homeostasis and produces oxidative stress that ultimately leads to cell damage and subsequent cell death. More ROS generation was observed at higher drug concentrations of sacubitrilat (Fig. 7).

The more uptake of DCFDA by cells leads to increased cell death. This could be due to the high sensitivity of SW-480 cells towards sacubitrilat. High ROS levels are prone to increase oxidative stress and induction of apoptotic-mediated cell death in cancer cells. A similar pattern of ROS generation was seen in SAHA-treated SW-480 cells. Likewise, the uptake of PI was observed in sacubitrilat and SAHA-treated cells (Fig. 6). In general, PI intercalates to the DNA bases of dead cells, with more PI-stained nuclei indicating a higher level of cell death in treated cells. The DCFDA and PI staining results indicate that sacubitrilat and SAHA play a role in apoptotic-mediated cell death in SW480 cells.

#### Role of sacubitrilat in cell cycle arrest in SW-480 cells

**Figure 8:**
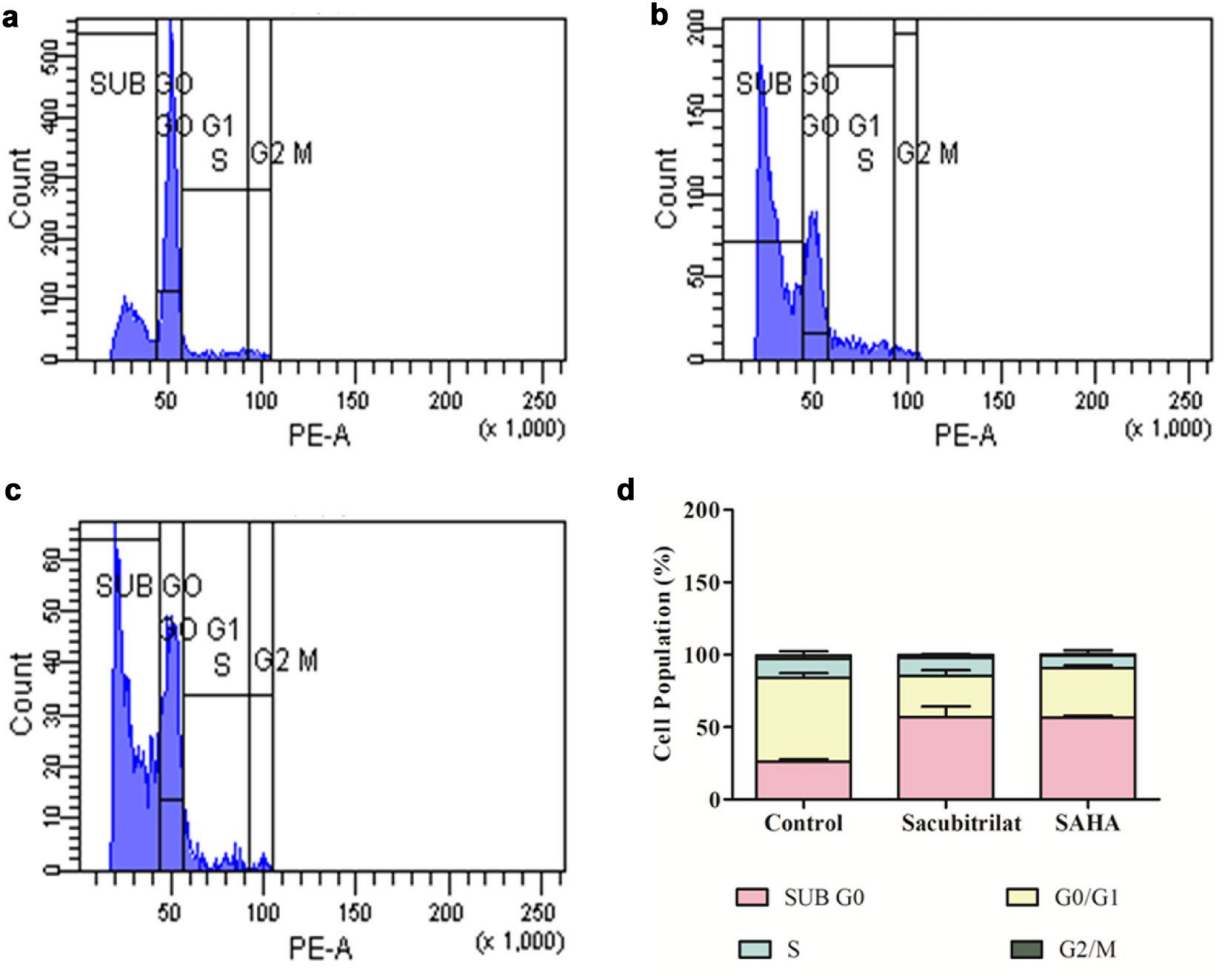
Effect of sacubitrilat on the cell cycle regulation in colorectal cancer cells; (**a**) Control cells (without treatment), (**b**) Sacubitrilat arrest cells at subG0/G1 phases of the cell cycle, (**c**) SAHA arrest cell cycle at subG0/G1 phases, (**d**) Calculated % of cells population in different phases of the cell cycle.

The effect of sacubitrilat on cell cycle regulation was investigated by PI staining using FACS analysis (Fig. 8). In the control sample, more than 97.6% of cells were present in the G0/G1 and S phases, which indicated normal cell cycle regulation (Fig. 8a). Whereas, sacubitrilat has arrested 64.2% of cells in the subG0 phase and 24.2% of cells in the G1 phase (Fig. 8b and d). Similar results were observed in SAHA-treated SW-480 cells where 58% and 35.8% of cells were arrested in subG0 and G1 phases, respectively (Fig. 8c,d). This suggested that sacubitrilat and SAHA have a similar mode of action against colon cancer in the regulation of the cell cycle (Fig. 8d).

**Figure 9:**
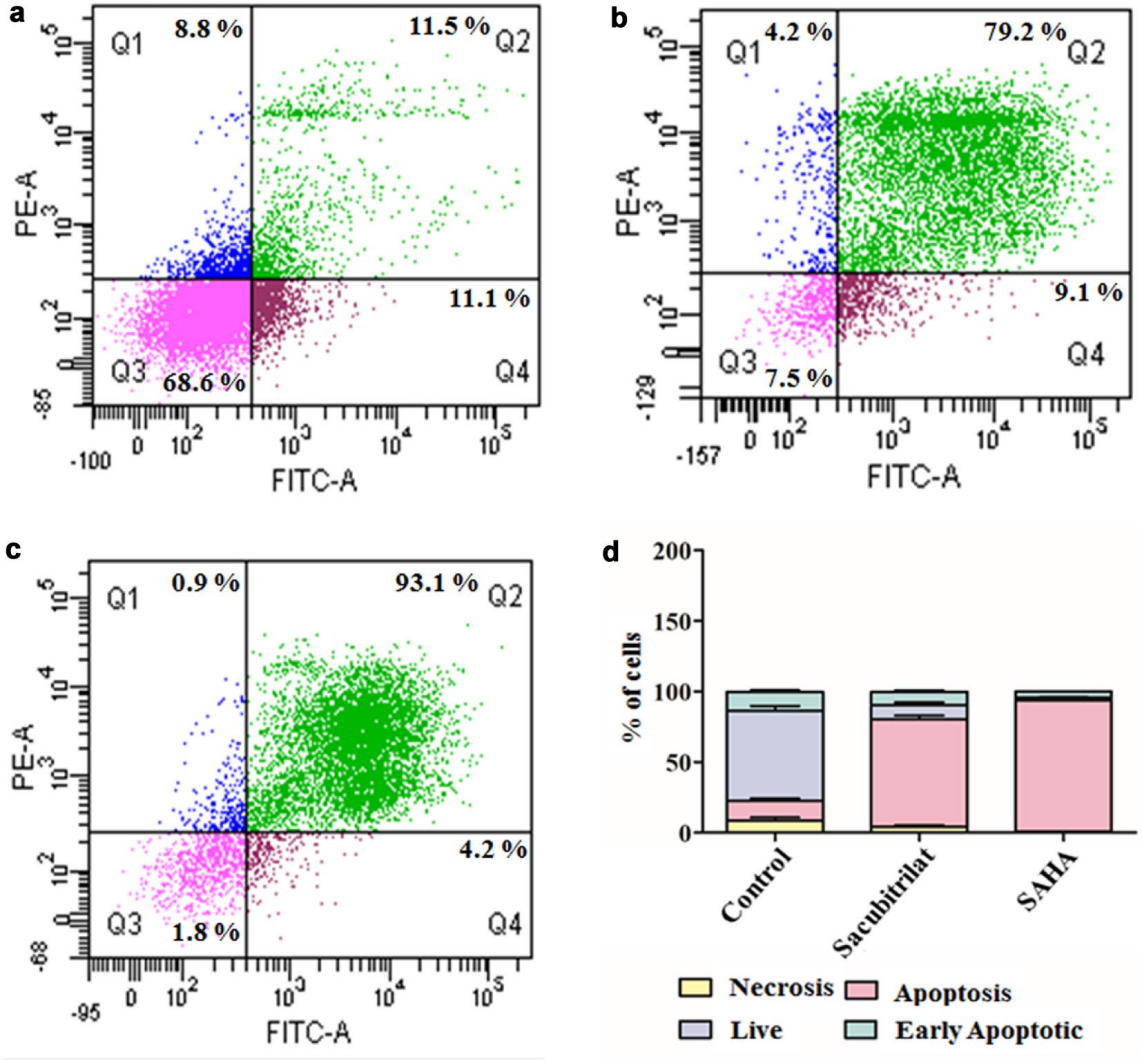
Apoptotic-mediated cell death in SW480 cells; (**a**) Control cells (without treatment), (**b**) Sacubitrilat induced apoptotic mediated cell death, (**c**) SAHA induced apoptotic-mediated cell death, and (**d**) Percent of cells arrested in SW-480 after the treatment of sacubtrilat and SAHA. (The bars represent the percentage of cells from Quadrant, Q1: Necrosis, Q2 = Apoptotic, Q3 = Live, and Q4 = Early Apoptotic cells).

In addition, Annexin V-FITC flow cytometry study was carried out to investigate the role of sacubitrilat in the induction of apoptotic-mediated cell death in SW-480 cells (Fig. 9). In the control sample, 68.6% of cells were alive, while sacubitrilat and SAHA-treated samples had 7.5% and 1.8% live cells, respectively (Fig. 9a). After 48 h of treatment, sacubitrilat induced 79.2% apoptotic-mediated cell death in SW-480, while 9.1% of cells were in the early apoptotic stage (Fig. 9b, d). The SAHA-treated cells have induced more apoptotic-mediated cell death (93.1%) in SW-480 (Fig. 9c, d). The FACS analysis and DCFDA/PI staining anticipated that sacubitrilat induces apoptotic-mediated cell death by generating high ROS and arresting the cell cycle at the subG0/G1 phases. Compared to SAHA, sacubitrilat showed a similar mode of action in ROS generation and cell cycle arrest. Hence, based on our findings, we can conclude that sacubitrilat has promising anticancer activity and may be used for the treatment of cancer.

#### Sacubitrilat downregulated the expression of epigenetic regulators (HDACs) in SW-480 cells

**Figure 10:**
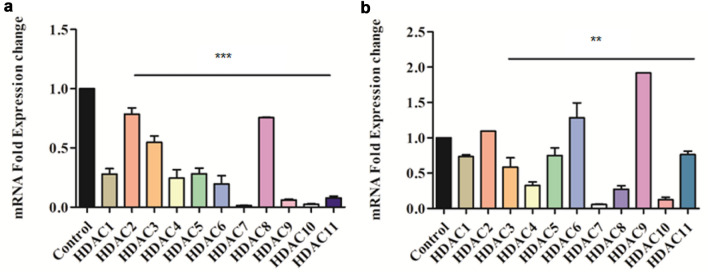
Effect of sacubitrilat modulated the expression profile of histone deacetylase enzymes in SW-480 cells.

The screening of the PLIP profile of FDA-approved HDAC inhibitor SAHA with LTA4H resulted in the identification of sacubitrilat. Therefore, herein, we have studied the role of sacubitrilat in the modulation of epigenetic regulators at the transcriptomic level (Fig. 10). In SW-480 cells, sacubitrilat inhibited the expression of class I, II and IV HDACs (Fig. 10a). Sacubitrilat remarkably (70–90% or 0.7–0.9 fold) downregulated the HDACs 1/3/4/5/6/7/9/10/11 while HDAC2 and HDAC8 were reduced slightly (22–25% or ~ 0.25 fold) as compared to the control.

On the same path, the SAHA was downregulating the expression of all HDACs except HDAC-2/6/9 (Fig. 10b). In comparison to SAHA, sacubitrilat plays a similar role in the modulation of epigenetic regulators (HDACs) in SW-480 cells. Therefore, sacubitrilat can be used as a potent HDAC inhibitor for the treatment of cancer. Also, sacubitrilat can be used for the treatment of neurodegenerative disorders, diabetes, learning-memory dysfunctions, and Huntington’s disease where HDACs play a critical role in their progression.

#### Sacubitrilat modulated the expression of epigenetic regulators and apoptosis markers at the proteomic level

**Figure 11:**
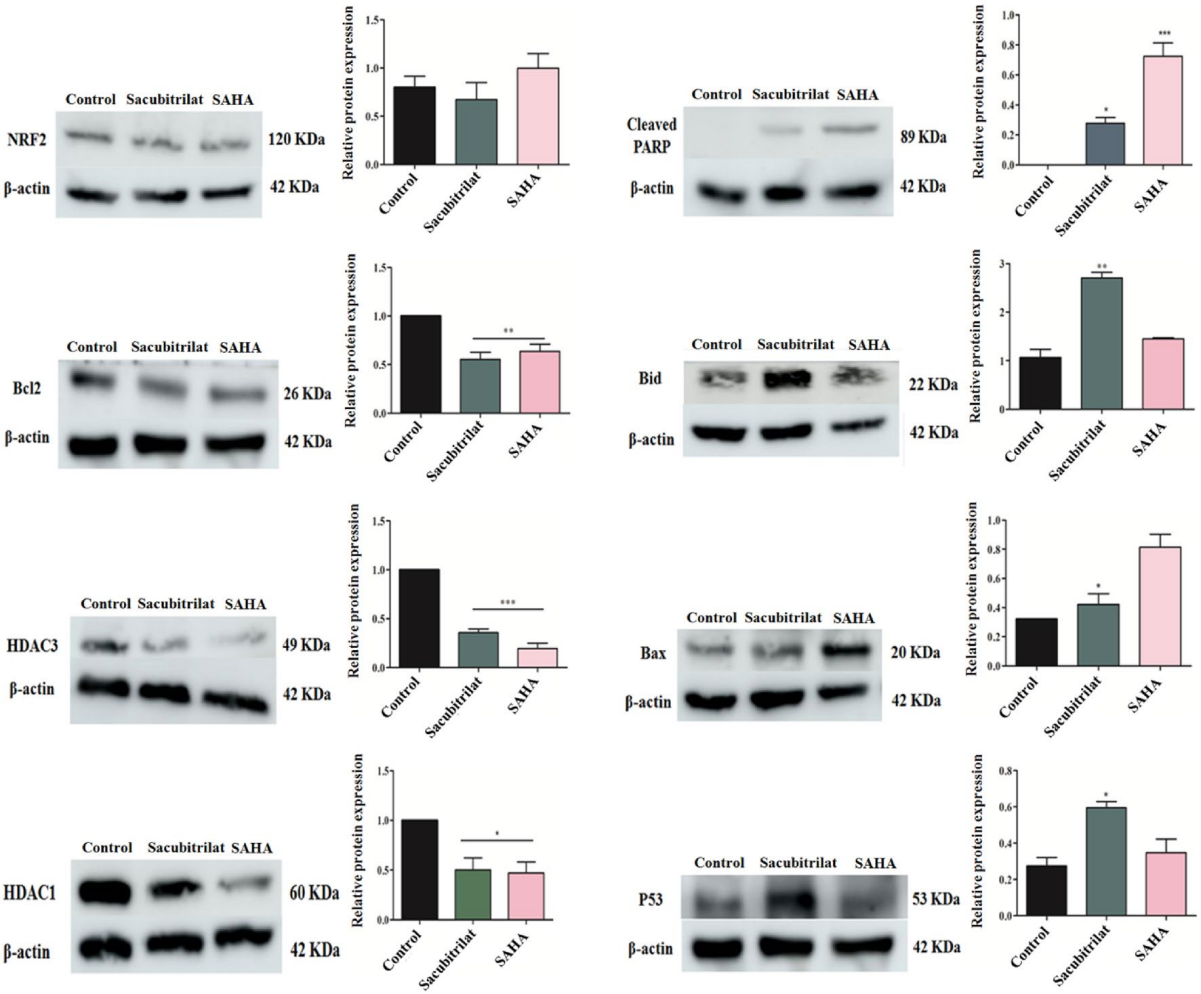
Sacubitrilat downregulated HDAC1, HDAC3 and anti-apoptotic Bcl2 and Nrf2 proteins, and upregulated the pro-apoptotic Bax, Bid, tumor suppressor p53, and generated cleaved PARP in colon cancer cells. The representative image of immunoblots is given while the graph displays a densitometry analysis of three immunoblots.

The role of sacubitrilat in the modulation of expression profiles of epigenetic regulators (HDAC isomers), pro-apoptotic proteins (Bax, Bid and PARP), tumor suppressor p53, anti-apoptotic protein-Bcl2 and transcription factor (Nrf2) were studied at a proteomic level using western blot analysis (Fig. 11). In SW-480 cells, sacubitrilat inhibited the expression of HDAC1 and HDAC3 by 0.5 and 0. sixfold as compared to untreated cells, respectively (Fig. 11). In contrast, sacubitrilat increased the protein expression level of tumor suppressor p53 and pro-apoptotic proteins such as Bax and Bid by 0.2–2.5 fold with respect to control cells. A reduction (0.2–0.25 fold) in the expression of anti-apoptotic protein-Bcl2 and transcription factor (Nrf2) was observed after the treatment of sacubitrilat. In addition, the cleavage of PARP was noticed in the sacubitrilat-treated SW-480 cells. The epigenetic regulators, pro- and anti-apoptotic markers and Nrf2 are associated with the severe pathogenesis in different cancer subtypes. The inhibition of expression of HDACs, up-regulation of p53, Bax, Bid, cleavage of PARP and downregulation of Bcl2 and Nrf2 by sacubitrilat in SW-480 cells help to discover the anticancer properties of sacubitrilat.

Our in-silico and in-vitro results support the repurposing of sacubitrilat as an anticancer agent by inducing apoptotic pathways that were generally modulated by the reference HDACi ‘SAHA’. This may also suggest a synergistic use of sacubitrilat and SAHA in designing combinatorial treatment approaches for cancer and other human diseases.

## Discussion

Millions of individuals worldwide are affected by different types of cancers^77^. The current course of treatment is costly and produces several adverse effects^78,79^. Therefore, there is a need to discover new medications to treat cancer. Drug repurposing is one of the cutting-edge strategies that focused on finding new indications for the already known clinically approved drugs^80^. Recent advancements in high-dimensional genomics, proteomics, and computational therapeutic approaches make DR an effective strategy for the treatment of cancer and other human diseases^81,82^. Earlier, cardiovascular drugs have been successfully repurposed against breast, colon, oesophageal, and gastric cancer^83^.

As we know that the high expression of HDACs is strongly correlated with the proliferation, migration, invasion and metastasis in cancer subtypes^9,10,13,84^. The overexpression of HDAC1 and HDAC3 promotes proliferation, differentiation and metastasis in colon and prostate cancer^85–88^. Therefore, HDACs have been more intensively studied for their clinical significance and to design a prominent therapeutic approach to treat human cancer^89^. The molecular interactions between SAHA and LTA4H were used to identify the sacubitrilat from the PLIP fusion fingerprint database. The sacubitrilat yielded the lowest energy docked conformation with LTA4H as compared to Vorinostat (Table 2). The molecular interactions observed in docked complexes were reported in earlier crystallographic data of standard HDAC inhibitors (Vorinostat, Trichostatin, Panobinostat, Quisinostat, and Recolinostat) with the different HDAC isoforms (Table 3). The catalytic tyrosine residue from the active site pocket of LTA4H was involved in hydrogen bonding interaction with sacubitrilat. This is similar to the co-crystal data, where the catalytic tyrosine residue from the HDAC isoforms participated in hydrogen bonding interactions with Vorinostat, Trichostatin, Panobinostat, Quisinostat, and Recolinostat (Table 3). This residue interaction has been reported as a crucial factor in the inhibition of the catalytic activity of HDACs^90^. Similarly, glycine and lysine residues were also found to be involved in hydrogen bonding interactions with sacubitrilat and reference HDACi such as Trichostatin and Recolinostat (Tables 2 and 3). However, the catalytic interaction of histidine with HDACi was not observed in the sacubitrilat and LTA4H docking complex. The phenylalanine residue from the active site pocket of LTA4H and HDAC isoforms participated in π-stacking interactions with sacubitrilat and reference HDACi (Tables 2 and 3). This interaction provides structural stability to the enzyme-inhibitor complex (Table 3).

**Table 3:** Catalytic residues from the favourable docked complexes and crystal structures of HDACs involved in hydrogen bonding, π-stacking, Zn^2+^-metal interactions and hydrophobic interactions with sacubitrilat and reference HDAC inhibitors.

Docked complexes of Sacubitrilat/BIR/B65/BDS with 4R7L	Vorinostat (4R7L/4LXZ/4QA0/4BZ6/5EEI)	Trichostatin (5EEF)	Panobinostat (5EF8)	Quisinostat (6HSK)	Recolinostat (5WGL)
Residues involved in Hydrogen bonds					
Gly268/269		Gly361			
Tyr378/383	Tyr308	Tyr363	Tyr745	Tyr306	Tyr745
Met564					
Lys565		Lys330			
	Asp98/104				
	His292/573	His193			His573/574
			Ser531		
Residues involved in π-stacking					
Phe314	Phe314	Phe202	Phe583/643	Phe152/180	
Tyr267					
Involved in Zn^2+^-metal interactions					
Yes	Yes	Yes	Yes	Yes	Yes
Residues involved in hydrophobic interactions					
Gln136,Ala137 Tyr267,Val292 His295,Trp311 Phe314,Pro374 Tyr378,Lys565	Trp311,Leu369 Pro374,Tyr378	His82, Pro83 Ser150,Gly201 Phe202,His232 Gu360,Trp261 Gly361,Asp32 3Gly362	Asp460,His46 His573,His574 Gly582,His614 Asp705,Leu712 Gly743	Asp101,His142 His143,Gly151 Gly206,Phe207 Pro209,Gly210 Leu274,Gly304	His462,Pro464 Ser531,Gly582 Phe643,Phe583His614,Asp705Pro711,Leu712Gly743

The sacubitrilat co-ordinated with Zn^2+^ in the active site pocket of LTA4H which is similar to the reference HDACi co-crystallized with different HDAC isoforms (Tables 1 and 3). This suggests the metal-dependent inhibition of LTA4H and HDACs by sacubitrilat and reference HDACi, respectively^90^. Additionally, the hydrophobic contacts observed in the docked and co-crystal complexes of sacubitrilat and HDACi may be playing a vital role in stabilizing the enzyme-inhibitor complexes. Hence, tyrosine, glycine, lysine and histidine residues from the active site pocket of HDACs seem to be crucial for substrate binding and catalytic inhibition. In addition, the Zn^2+^ coordination with inhibitors suggests the metal-dependent inhibition of HDACs. The tyrosine, histidine, phenylalanine and hydrophobic residues were observed in hydrogen bonding, π-stacking and hydrophobic interactions with sacubitrilat and other reference HDAC inhibitors such as SAHA, trichostatin, panobinostat and quisinostat (Table 3). All these non-covalent interactions are governed by the presence of pharmacophore features (HBA, HBD, RA and HYP) in the pharmacophore models generated by using HDACs inhibitors (Supplementary Table 1). Hence, it is speculated that sacubitrilat may be manifesting its anti-cancer effect similar to SAHA by inhibiting tumor growth, reducing HDAC expression, and inducing cell cycle arrest and apoptosis in cancer cells^91–93^.

The sacubitrilat inhibited the growth of colorectal and breast cancer at 14.07 µg/mL and 23.02 µg/mL (Fig. 3). The treated SW-480 cells have reduced the migration potential up to 70–80% in comparison to migration observed in control cells (Fig. 5). The increased ROS generation was observed in cancer cells at higher concentrations of sacubitrilat and SAHA (Figs. 6 and 7). Similar to this, a decrease in cell viability and a significant increase in ROS production were observed in SAHA-treated colon cancer cells^92–94^. The higher uptake of DCFDA and PI in treated cells has suggested that sacubitrilat and SAHA induced apoptotic-mediated cell death, as observed in earlier studies^94^. FACS analysis showed that sacubitrilat and SAHA arrested the cell cycle at subG0/G1 phases, this anticipated the inhibition of cell proliferation and differentiation in SW-480 cells (Fig. 8). In addition, sacubitrilat downregulated Class I, II and IV HDACs at the transcriptomic level, and reduced HDAC1 and HDAC3 at the proteomic level (Figs. 10 and 11). These results are comparable to earlier studies where SAHA inhibited the growth of colon tumors by reducing the expression of HDAC1/2/3/4^89,93^. The downregulation of tumor suppressor p53, pro-apoptotic Bax and Bid, and upregulation of anti-apoptotic Bcl2 and Nrf2 are significantly attributed to the progression and metastasis of different cancer subtypes^95,96^. The elevated level of Nrf2 promotes cell proliferation in colon cancer, induces transcription of anti-apoptotic protein Bcl2 and impaired the expression of p53^96,97^. In this study, the pro-apoptotic markers (p53, Bax, Bid and PARP) were upregulated and anti-apoptotic markers (Bcl2 and Nrf2) were downregulated after the treatment of sacubitrilat and SAHA in colon cancer (Fig. 11). This clearly outlines the potential of sacubitrilat to induce apoptotic-medicated cell death in SW-480 colon cancer cells. Similar results have also been observed in the colon, prostate carcinoma and hepatocellular carcinoma after the treatment of SAHA and Valproic acid^91,93,98^. In general, the activation of tumor-suppressor p53 induces cell cycle arrest and apoptotic-mediated cell death to control the cancer growth, progression and metastasis in various cancer subtypes by up-regulating pro-apoptotic Bax protein and downregulating anti-apoptotic Bcl-2 protein^99,100^. However, the expression of mutated p53 has been associated with increased tumor progression in various cancer subtypes^101,102^. Interestingly, the pharmacological reactivation of mutant p53 by small molecules (PRIMA, MIRA-1 and Cisplatin) showed antitumor activity in colon cancer and other human tumors^103–110^. Moreover, our transcriptomic and proteomic results concord with earlier reports where SAHA has been reported to increase the expression of p53, Bax and cleaved Parp proteins while decreasing the expression of HDACs1/2/3/4/5/6/7/8 and Bcl-2 proteins which controlled the growth of colon cancer by inducing apoptotic-mediated cell death^89,111,112^. Therefore, the increased expression of mutated p53 after the treatment of sacubitrilat might be associated with growth inhibition of SW-480 colon cancer cells. All these preceding literature reports and our in-silico and in-vitro results strongly suggested that the mode of action of sacubitrilat and SAHA as anticancer agents is the same. Additionally, the sacubitrilat is an active metabolite of sacubitril, which demonstrated potent anti-hypertensive and anti-diabetic activity^113–115^. Therefore, sacubitrilat may also be used for the treatment of cancer patients having heart-related and diabetic complications. Hence, performed studies may help to design and use suitable therapeutic approaches to treat cancer and other human diseases by considering the synergetic inhibitory effect and mechanism of sacubitrilat and SAHA.

## Conclusion

Herein, we have repurposed the neprilysin inhibitor "Sacubitrilat" as an anticancer agent by modulating the expression profile of epigenetic regulators and apoptosis markers. The screening of the PLIP fusion fingerprint database using PLIP profiles of SAHA with LTA4H and panobinostat with HDAC6 resulted in 17 FDA-approved, experimental and investigational drugs. Molecular docking was assisted to identify the five best-hit compounds based on the better energy profile than the reference drug SAHA. Sacubitrilat demonstrated potent anticancer activity against colorectal cancer and TNBC by inhibiting cell proliferation at IC_50_ = 14.07 μg/mL and IC_50_ = 23.02 μg/mL, respectively. Sacubitrilat inhibited cell migration, increased ROS generation, arrested the cell cycle at subG0/G1 phases and induces apoptotic-mediated cell death in SW-480 cells. In addition, sacubitrilat downregulated HDAC isomers at the mRNA level, while the expression of HDAC1 and HDAC3 reduced at the proteomic level. The up-regulation of tumor suppressor p53 and pro-apoptotic markers (Bax, Bid and PARP) and down-regulation of anti-apoptotic (Bcl2 and Nrf2) confer the apoptotic-mediated cell death induced by sacubitrilat in SW-480 cells. The anticancer potential of sacubitrilat is comparable to the SAHA (Reference HDACi). Our in-silico and in-vitro studies supported the repurposing of neprilysin inhibitor ‘Sacubitrilat’ as a potential anti-cancer agent against colorectal cancer. However, further pre-clinical and clinical studies are required to ascertain the mechanistic role of sacubitrilat against colorectal cancer.

## Supplementary Information

Supplementary Information.

## Data Availability

The datasets used and/or analysed during the current study are available from the corresponding author upon reasonable request.

## Abbreviations

6LD: Sacubitrilat
B65: (1R)-4-(3-phenoxyphenyl)-1-phosphonobutane-1-sulfonic acid
BDS: 2,3-Bis-benzo[1,3]dioxol-5-ylmethyl-succinic acid
BIR: N-[3-[(1-Aminoethyl)(hydroxy)phosphoryl]-2-(1,1′-biphenyl-4-ylmethyl)propanoyl]alanine
NPV: 4-[8-(3-Nitrophenyl)-1,7-naphthyridin-6-yl]benzoic acid
MTT: 3-[4,5-Dimethylthiazol-2-yl]-2,5 diphenyl tetrazolium bromide
6TJ: 3-Oxidanylidene-2-phenyl-1~{H}-isoindole-4-carboxylic acid
7CA: 5,7-Dihydroxy-2-(4-methoxyphenyl)-8-(3-methylbutyl)-4-oxo-4h-chromen-3-Yl 6-deoxy-alpha-L-mannopyranoside
CRI: 5-(4-Methyl-benzoylamino)-biphenyl-3,4′-dicarboxylic acid 3-dimethylamide-4′-hydroxyamide
HPI: N-(1-Carboxy-3-phenylpropyl)phenylalanyl-alpha-asparagine
792: N-{[4-(but-2-yn-1-yloxy)phenyl]sulfonyl}-5-methyl-D-tryptophan
AP5: Bis(adenosine)-5′-pentaphosphate
AXA: (5S)-5-(2-amino-2-oxoethyl)-4-oxo-N-[(3-oxo-3,4-dihydro-2H-1,4-benzoxazin-6-yl)methyl]-3,4,5,6,7,8-hexahydro[1]benzothieno[2,3-d]pyrimidine-2-carboxamide
DPS: 3-(1h-Indol-3-Yl)-2-[4-(4-phenyl-piperidin-1-Yl)-benzenesulfonylamino]-propionic acid
T5A: P1-(5′-Adenosyl)P5-(5′-thymidyl)pentaphosphate
TXC: 2-[(2-Amino-6-oxo-1,6-dihydro-9H-purin-9-yl)methoxy]ethyl L-valinate
YE7: Imidazo[2,1-A]isoquinoline-2-carbohydrazide
Z5A: P1-(5′-Adenosyl)P5-(5′-(3′azido-3′-deoxythymidyl))pentaphosphate
FACS: Fluorescence-activated cell sorting
qRT-PCR: Real-time quantitative reverse transcription PCR
PLIP: Protein–ligand interaction profiler
FDA: Food and drug administration
HDAC: Histone deacetylases
Nrf2: Nuclear factor erythroid 2–related factor 2
PARP: Poly (ADP-ribose) polymerases
NAD: Nicotinamide adenine dinucleotide
HDACi: HDAC inhibitors
DR: Drug repurposing
QSAR: Quantitative structure–activity relationships
MD: Molecular dynamics
VEGFR2: Vascular endothelial growth factor receptor
FabI: Enoyl-ACP reductase
SAHA, SHH: Suberoylanilide hydroxamic acid
PDB: Protein data bank
CM-H_2_ DCFDA: 2′,7′-Dichlorodihydrofluorescein diacetate
HRP: Horseradish peroxidase
DMSO: Dimethyl sulfoxide
DMEM: Dulbecco’s modified eagle media
FBS: Fetal bovine serum
ROS: Reactive oxygen species
DCF: 2′,-7′-Dichlorofluorescein
PI: Propidium iodide
Cdna: Complementary DNA
WBA: Western blot analysis
RIPA: Radioimmunoprecipitation assay
PVDF: Polyvinylidene fluoride
SDS-PAGE: Sodium dodecyl-sulfate polyacrylamide gel electrophoresis
TBST: Tris-buffered saline containing 0.1% Tween-20
ANOVA: Analysis of variance
LTA4H: Leukotriene A4 hydrolase
TNBC: Triple negative breast cancer
COVID: Coronavirus disease
PBS: Phosphate buffer saline
FITC: Fluorescein isothiocyanate
GAPDH: Glyceraldehyde-3-phosphate dehydrogenase
TTD: Therapeutic target database
IPs: Interaction patterns
