# Supplementary material for: Repurposing of neprilysin inhibitor ‘sacubitrilat’ as an anti-cancer drug by modulating epigenetic and apoptotic regulators
Source: Sci Rep. 2023 Jun 19;13:9952. doi: 10.1038/s41598-023-36872-0 (PMC10279647; doi:10.1038/s41598-023-36872-0)
Supplement: Supplementary file 1 — Supplementary Information. [file 41598_2023_36872_MOESM1_ESM.docx]

**Supplementary Information**

**Repurposing of neprilysin inhibitor ‘Sacubitrilat’ as an anti-cancer drug by modulating epigenetic and apoptotic regulators**

Navanath Kumbhar,^1#^ Snehal Nimal,^1#^ Deeksha Patil,^2^ Florian Kaiser, V.^3^ Joachim Haupt,^3^ Rajesh Gacche.^1*^

1. Department of Biotechnology, Savitribai Phule Pune University Pune, Maharashtra (MS),

411007, India.

2. Department of Microbiology, Savitribai Phule Pune University Pune, Maharashtra (MS),

411007, India.

3. PharmAI GmbH, Tatzberg 47, 01307 Dresden, Germany.

# Navanath Kumbhar and Snehal Nimal equally contributed to the work

***Corresponding Address:**

Professor Rajesh N Gacche,

Department of Biotechnology,

Savitribai Phule Pune University (SPPU), Pune, India, 411007.

Email.: [rngacche@unipune.ac.in](mailto:rngacche@unipune.ac.in), [rngacche@rediffmail.com](mailto:rngacche@rediffmail.com)

Tel.: +91-20-25694952

Fax.: +91-20-25692248

**Raw Data of Western blots**

After we observed the equal β-actin, we cut the original blot into four different blots to check the expression level of proteins having different molecular weights at a time. The full blots for β-actin, HDAC1, HDAC3, Bcl2, Nrf2, p53, Bax, Bid and Parp proteins have been included here. The proteins HDAC1, Nrf2, p53 and Parp have a molecular weight higher whereas HDAC3, Bcl2, Bax and Bid have lower molecular weight as compared to β-actin.

**
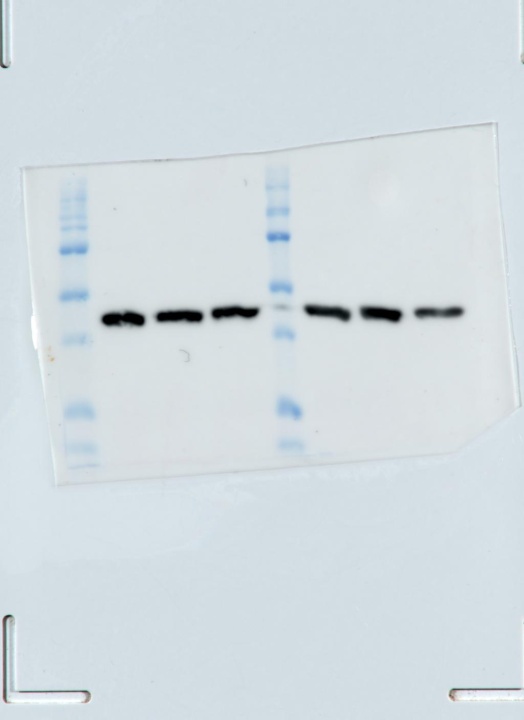

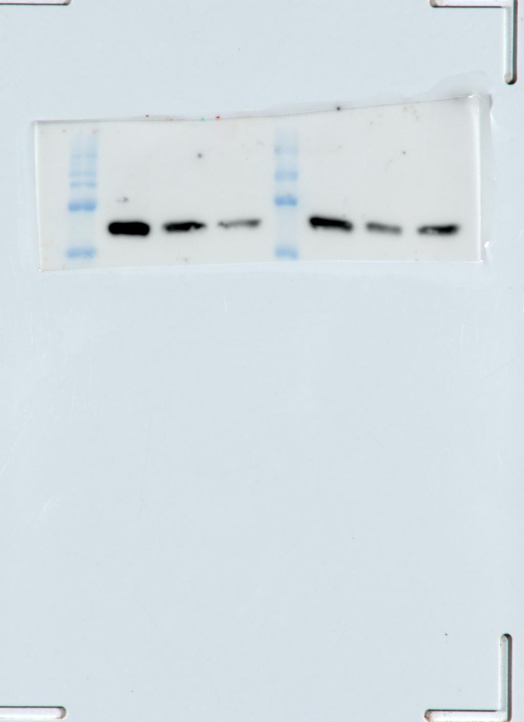
**

**β-actin HDAC-1**

**
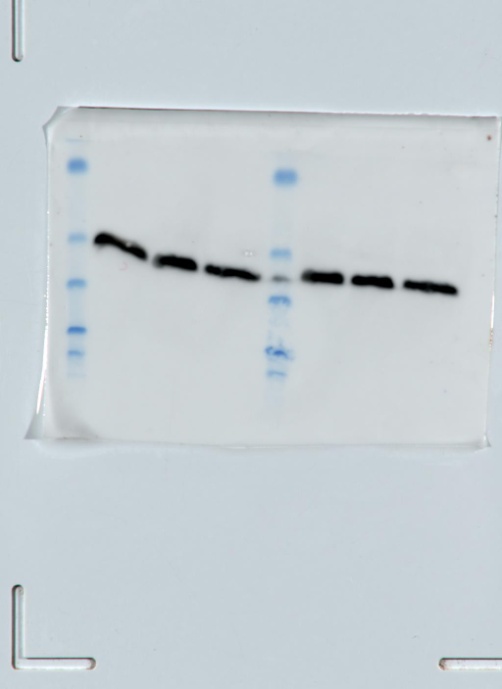

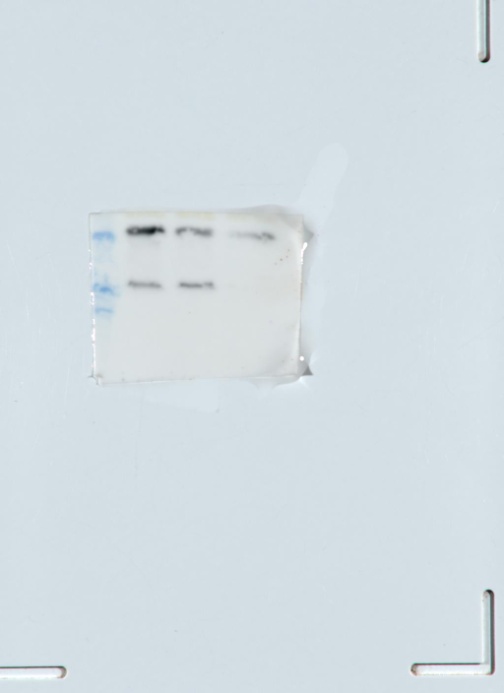
**

**β-actin HDAC-3**

**
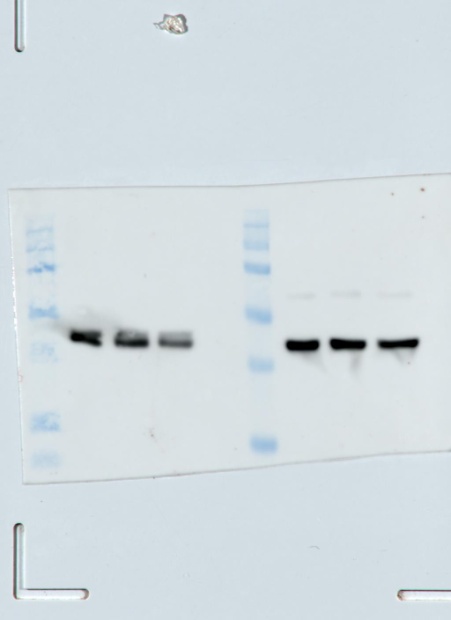

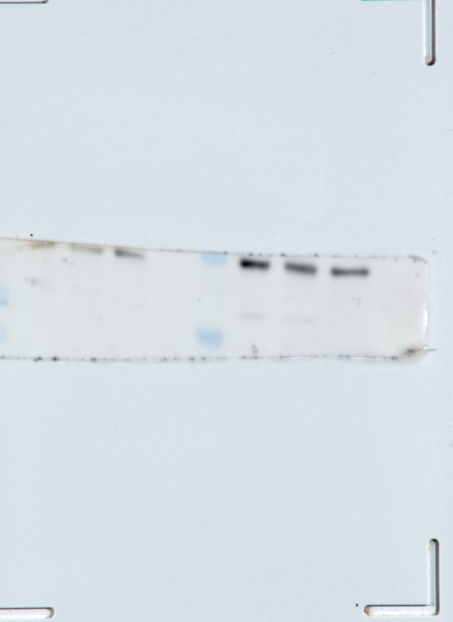
**

**β-actin Bcl2**

**
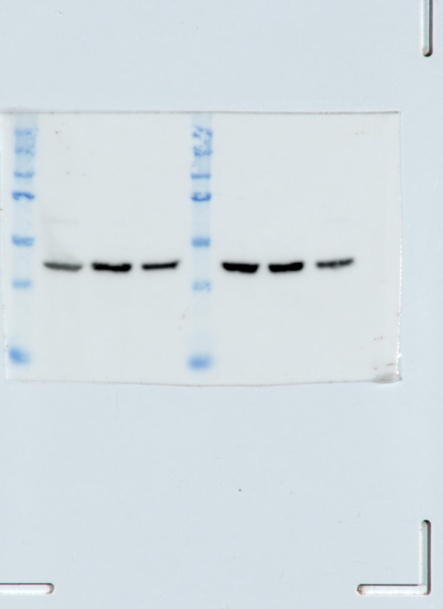

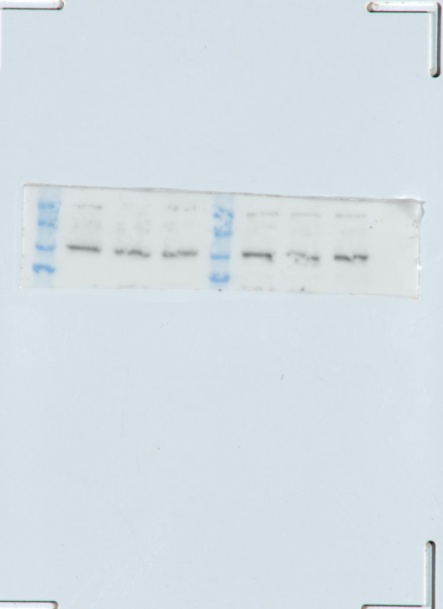
**

**β-actin Nrf2**

**
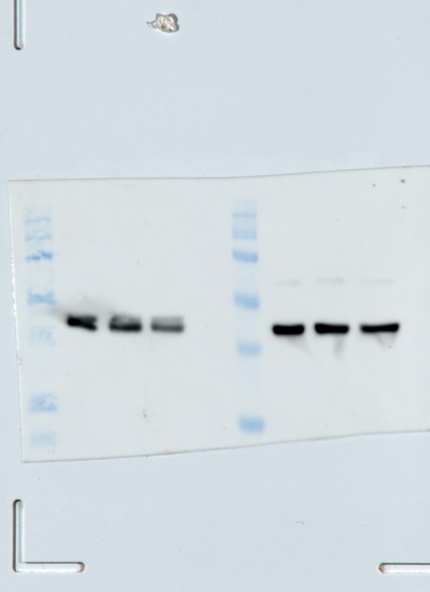
**
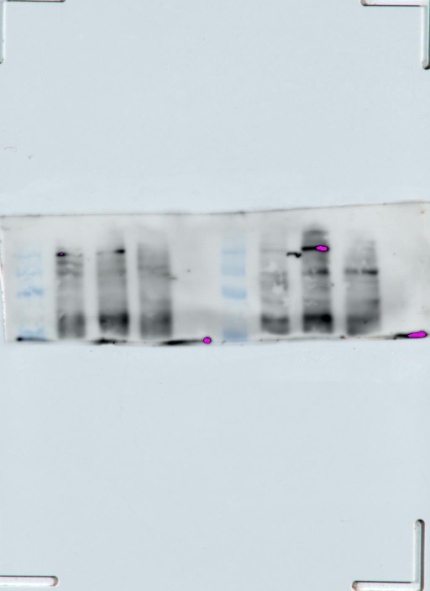


**β-actin p53**

**
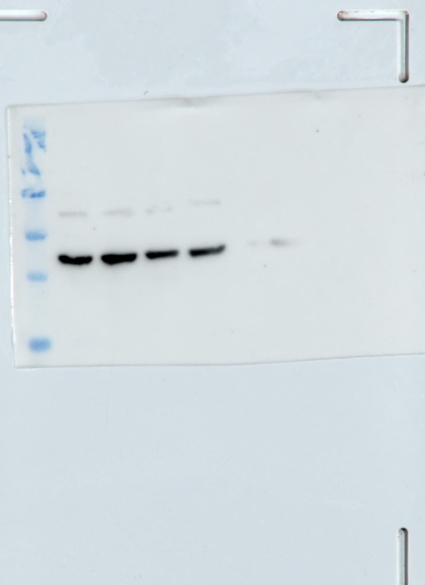
** **
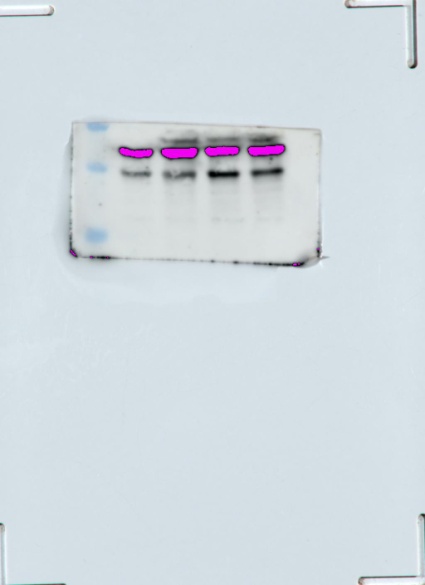
**

**β-actin Bax**

Note: The upper saturated band is β-actin while below is Bax proteins.

**
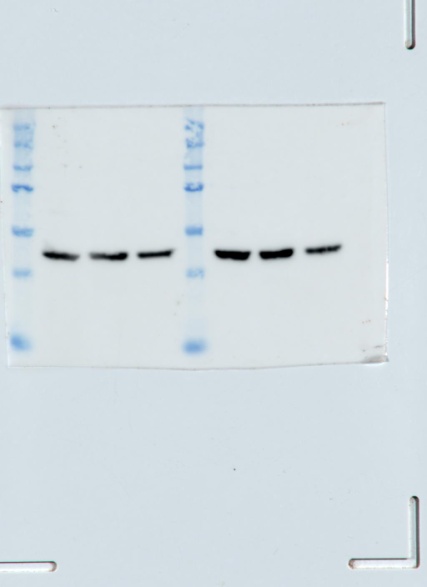
**
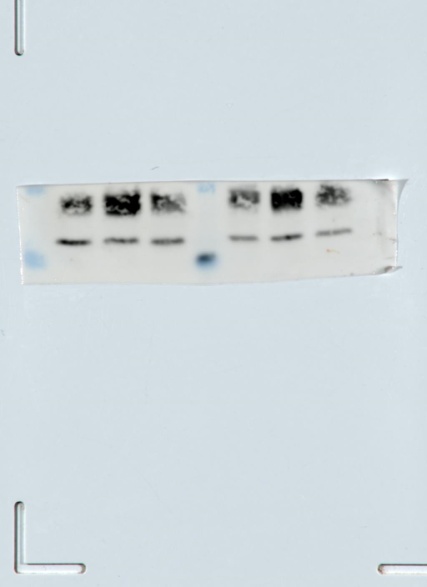


**β-actin Bid**


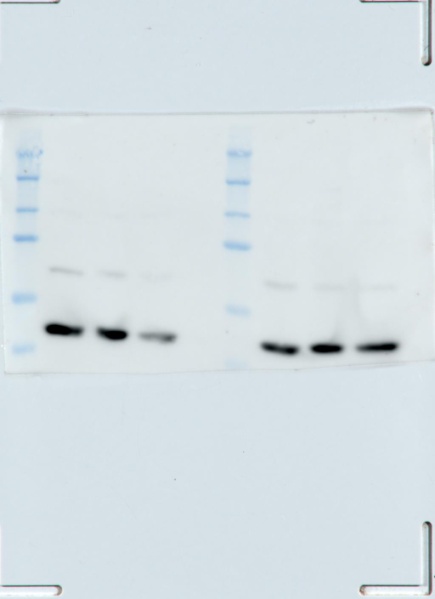

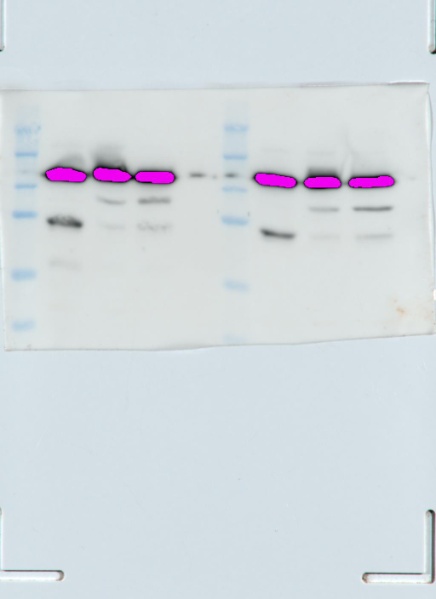


**β-actin Parp**

**Table 1SI:** Predicted pharmacophore features for HDACs inhibitors with differnet methods.

| **HDACs isoforms** | **Pharmacophore Features** | **Method** | **References** |
| --- | --- | --- | --- |
| HDAC1 | HBA, HBD, RA | 3D-QSAR modeling | 1 |
| HDAC1 | HBA, RA, 2HYP | Ligand-based pharmacophore model | 2 |
| HDAC2 | HBA, HBD, RA, HYP | Ligand-based pharmacophore model | 3 |
| HDAC3 | HBA, HYP, HYP, RA | 3D-QSAR pharmacophore modeling | 4 |
| HDAC6 | HBD, RA, 2HYP | Structure based pharmacophore modeling | 5 |
| HDAC6 | HBA, HBD, RA, HYP | Ligand-based pharmacophore model | 6 |
| HDAC8 | HBA, 2HBD, HYP | Ligand-based pharmacophore model | 7 |
| HDAC | HBA, HBA, HBD, HYP | Ligand based Pharmacophore modeling | 8 |

**References:**

1. Sirous, H. et al. Computer-Driven Development of an in Silico Tool for Finding Selective Histone Deacetylase 1 Inhibitors. *Molecules* **25,** 1952 (2020).
2. Liqin,Y. U. Fei, L. I. U. Yadong, C. & Qidong, Y. Pharmacophore Identification of Hydroxamate HDAC 1 Inhibitors. *Chin J Chem* **27**, 557-564 (2009).
3. Kandakatla, N. & Ramakrishnan, G. Ligand Based Pharmacophore Modeling and Virtual Screening Studies to Design Novel HDAC2 Inhibitors. *Adv Bioinform.* **ID 812148,** 11 (2014).
4. Kumbhar N, Nimal S, Barale S, Kamble S, Bavi R, Sonawane K, Gacche R. Identification of novel leads as potent inhibitors of HDAC3 using ligand-based pharmacophore modeling and MD simulation. Sci Rep. 2022 Feb 2;12(1):1712. doi: 10.1038/s41598-022-05698-7. PMID: 35110603; PMCID: PMC8810932.
5. Wang, Y. et al. Hierarchical virtual screening of the dual MMP-2/HDAC-6 inhibitors from natural products based on pharmacophore models and molecular docking. *J Biomol Struct Dyn.* **37(3)**, 649-670 (2019).
6. Zeb, A. et al. Investigation of non-hydroxamate scaffolds against HDAC6 inhibition: A pharmacophore modeling, molecular docking, and molecular dynamics simulation approach. *J Bioinform Comput Biol.* **16(3)**, 1840015 (2018).
7. Thangapandian, S. et al. Docking-enabled pharmacophore model for histone deacetylase 8 inhibitors and its application in anti-cancer drug discovery. *J Mol Graph Model* **29(6)**, 894 (2011).
8. Liu, J. et al. Combined pharmacophore modeling, 3D-QSAR and docking studies to identify novel HDAC inhibitors using drug repurposing. *J Biomol Struct Dyn.* **38(2)**, 533-547 (2020).
